# Exploring the Chemical Features and Biomedical Relevance of Cell-Penetrating Peptides

**DOI:** 10.3390/ijms26010059

**Published:** 2024-12-25

**Authors:** Liliana Marisol Moreno-Vargas, Diego Prada-Gracia

**Affiliations:** Research Unit on Computational Biology and Drug Design, Children’s Hospital of Mexico Federico Gómez, Mexico City 06720, Mexico

**Keywords:** cell-penetrating peptides, physical–chemical properties, cellular uptake, cargo delivery, prediction of CPPs

## Abstract

Cell-penetrating peptides (CPPs) are a diverse group of peptides, typically composed of 4 to 40 amino acids, known for their unique ability to transport a wide range of substances—such as small molecules, plasmid DNA, small interfering RNA, proteins, viruses, and nanoparticles—across cellular membranes while preserving the integrity of the cargo. CPPs exhibit passive and non-selective behavior, often requiring functionalization or chemical modification to enhance their specificity and efficacy. The precise mechanisms governing the cellular uptake of CPPs remain ambiguous; however, electrostatic interactions between positively charged amino acids and negatively charged glycosaminoglycans on the membrane, particularly heparan sulfate proteoglycans, are considered the initial crucial step for CPP uptake. Clinical trials have highlighted the potential of CPPs in diagnosing and treating various diseases, including cancer, central nervous system disorders, eye disorders, and diabetes. This review provides a comprehensive overview of CPP classifications, potential applications, transduction mechanisms, and the most relevant algorithms to improve the accuracy and reliability of predictions in CPP development.

## 1. Introduction

Cell-penetrating peptides (CPPs) are small peptides, either synthetic or natural, consisting of 4 to 40 amino acids. They possess a net positive charge at a physiological pH and, unlike most peptides, exhibit the remarkable ability to cross cell membranes while preserving the functional integrity of the cargo they transport [1]. Since the pioneering discovery of CPPs over thirty years ago [2,3], these peptides have found diverse applications. CPPs can serve as vectors for delivering various payloads, including small peptides, full-length proteins, nucleic acids (such as RNA and DNA), liposomes, nanoparticles, and viral particles, as well as radioisotopes and other fluorescent probes [4,5]. Importantly, CPPs are not limited to acting solely as carriers for functional peptides into the cell interior; they can also incorporate functional motifs themselves [6,7].

CPPs exhibit a diverse range of cationic, anionic, and neutral sequences, displaying varying degrees of hydrophobicity and polarity. These characteristics are influenced by the amino acid composition and three-dimensional (3D) structure of the CPPs. While CPPs, in general, lack sequence identity, certain groups of CPPs share high-sequence homology and common structural features. The specific modes and extents of cellular uptake are determined not only by the structural diversity of CPPs but also by the nature of the cargo they carry, whether it is covalently or non-covalently attached. This cargo attachment profoundly affects the transport of CPPs across the cell membrane.

The cell membrane serves as a barrier to the intracellular delivery of potential diagnostic or therapeutic cargoes. The cellular uptake of CPPs involves various pathways, including macropinocytosis, caveolae and/or lipid raft-mediated endocytosis, as well as those mediated by membrane proteins packaged in clathrin-coated vesicles [8,9,10]. Cells continuously internalize and recycle one to five times the equivalent of their cell surface area hourly [11,12]. This continuous internalization process likely facilitates the entry of peptides with a strong affinity for the membrane through at least some endocytic pathways. Alternatively, CPPs with specific physicochemical properties may be capable of directly translocating across the cell membrane, similar to small molecules. However, the cell membrane is not a homogeneous double layer; certain regions exhibit higher density, while others are more fluid, depending on their lipid composition and density [13,14]. In turn, the lipid composition, density, and dynamics vary depending on the cell type, specific membrane region, and various signaling pathways [15,16,17]. This heterogeneity gives rise to different levels and modes of CPP uptake [18].

The uptake mode of several cationic CPPs varies depending on the CPP concentration. When the concentration exceeds a certain limit, rapid cytosolic uptake occurs, indicating direct translocation. At lower concentrations, the main uptake mechanism is primarily endocytic. The concentration limit differs for each CPP but is typically in the micromolar (μM) range. Studies conducted on CHO-K1 cells have shown that Penetratin translocation only occurs below 2 μM [19], suggesting that the cell type and membrane composition influence the balance between different entry pathways. Many cationic CPPs interact electrostatically with glycosaminoglycans (GAGs) on the cell surface as the initial step for cellular entry [20]. This interaction induces the clustering of GAGs on the cell surface, activating intracellular signals that facilitate cell entry through various internalization pathways, including direct translocation and endocytosis. For example, the activation of acid sphingomyelinase, followed by a change in the lipid composition of the cell membrane, facilitates the direct translocation of cationic CPPs [21].

Similar to most peptides, many CPPs exhibit poor absorption, distribution, metabolism, and excretion (ADME) properties, including rapid renal clearance, short half-life, low permeability, and occasionally low solubility [22]. Enhancing the pharmacological properties of CPPs involves strategies to increase permeability, reduce proteolysis and renal clearance, and prolong their half-life. Proteolysis can be reduced by stabilizing the 3D structure with unnatural amino acids, while renal clearance can be addressed by lowering free peptide concentrations through depot formation or carrier protein association [23].

The development of peptide drugs presents challenges but also holds promising prospects for targeted drug delivery due to their high specificity, selectivity, small sizes, ease of modification, and high biocompatibility. Incorporating CPPs into drug design provides an opportunity to address biological targets that would otherwise be difficult to target using small molecules. Over a quarter of a century since their discovery, refining the design of CPPs involves improving loading strategies, enhancing our understanding of how these peptides traverse cell membranes, and gaining deeper insights into the mechanisms that govern specificity in recognizing their molecular targets. This review highlights some of the most notable examples of CPPs, demonstrating their significant potential as delivery vehicles for in vivo applications. Special emphasis is placed on evaluating their structural and physicochemical characteristics.

### Overview of CPPs Classification

The initial and simplest classification of CPPs was based on their origin, categorizing CPPs into protein-derived and synthetic peptides. The sequences of the first CPPs were found in proteins from various origins and functions, and were accordingly named membrane translocation sequences (MTSs), protein transduction domains (PTDs), and Trojan peptides based on their respective roles. The identification and characterization of early protein-derived CPPs, such as the Tat peptide from the transactivator protein of HIV-1 transcription and the Penetratin from the homeotic transcription factor of Antennapedia, established the foundation for classifying peptides responsible for protein translocation.

The second classification of CPPs reflects advancements in peptide design, introducing chimeric peptides. Chimeric peptides are combinations of two or more different natural protein sequences or a combination of natural and/or synthetic peptide sequences. Various combinations of cationic, amphipathic, and hydrophobic sequences have led to the development of numerous delivery constructs. For instance, a potent secondary amphipathic peptide was designed for siRNA delivery into mammalian cells [24]. In another study, a series of steric blocking peptide nucleic acid oligonucleotides conjugated to an 18-mer PNA705 model oligonucleotide demonstrated enhanced cell-penetrating activity [25]. Additionally, an effective and versatile vehicle for the intracellular delivery of pDNA, SCO, and siRNA into various adherent and suspension cells was developed without cytotoxic side effects [26].

While these classifications provide insight into the relevance of cellular penetration, they do not correlate the properties of CPPs with their mechanisms of interaction with cell membranes or their modes of action. Therefore, it is essential to group CPPs based on their physicochemical features, as these features ultimately determine the effectiveness of their interactions and the potency of their effects.

**Figure 1:**
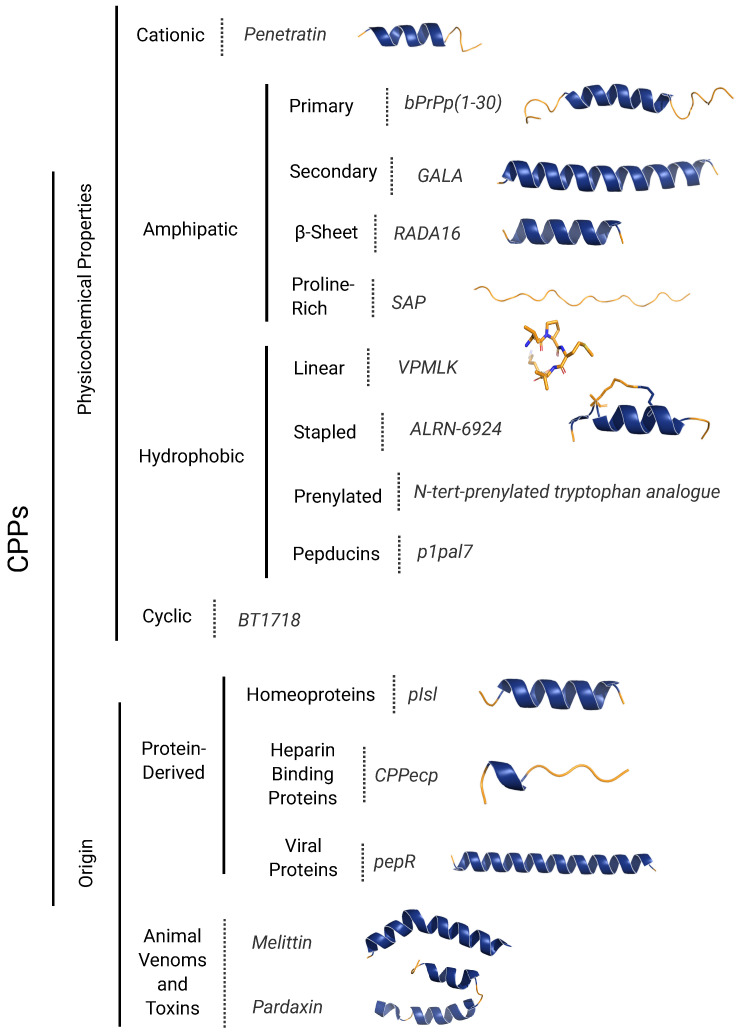
Classification of cell-penetrating peptides (cpps) by physicochemical properties and origin, with representative peptides for each category. This figure displays a selection of structurally diverse CPPs, classified into cationic (Penetratin, PDB ID: 1OMQ), amphipathic (Primary: bPrPp(1–30), PDB ID: 1SKH; Secondary: GALA; beta-sheet: RADA16; Proline-rich: SAP), hydrophobic (Linear: VPMLK (V5 antiapoptotic pentapeptide); Stapled: ALRN-6924, PDB ID: 8GJS; Prenylated: N-tert-prenylated tryptophan analogue [27]; Pepducins: p1pal-7 [28]) and cyclic (BT1718 [29]) peptides, as well as those derived from proteins (Homeoproteins: pIsl peptide; Heparin-binding proteins: CPPecp; Viral proteins: pepR), animal venoms, and toxins (Melittin (PDB ID: 6O4M) and, Pardaxin (PDB ID: 2KNS)). Each peptide is represented in its 3D conformation to enable the comparative visualization of structural motifs characteristic of each CPP type, which may impact their mechanisms of cellular uptake. AlphaFold [30] and PEP-FOLD3 [31] were used to predict the structures for those peptides without PDB ID. Notably, these configurations do not depict the spatial orientations involved in membrane interactions or the factors determining intracellular delivery efficiency.

To advance the understanding of CPPs, it is essential to classify them based on their physicochemical properties and origin, particularly in light of current developments in CPP design and application. This classification framework sheds light on the molecular mechanisms that govern CPP interactions with cellular membranes, including specific translocation pathways, binding affinities, and cellular uptake efficiencies, which are fundamental to optimizing their use as delivery vectors. CPPs are broadly categorized by their charge, hydrophobicity, and structural sequence motifs, characteristics that significantly impact their modes of cellular entry, endosomal escape, and cargo-release efficacy. Additionally, the origin of CPPs, whether derived from protein sequences, synthetic constructs, or biological sources such as viral proteins and animal toxins, further elucidates their functional adaptability and specificity for various therapeutic applications. Figure 1 provides a structured representation of CPP classifications, identifying key peptides within each category. This detailed categorization supports a more granular analysis of CPP properties, underscoring the diverse design strategies available to customize CPPs for targeted biomedical interventions, as will be elaborated in the following sections.

## 2. The CPPs Classification by Their Physicochemical Properties

CPPs can be classified into three categories based on their physicochemical properties, including charge, hydrophobicity, and the distribution of these descriptors along the peptide sequence. Although CPPs exhibit a wide range of sequences, three main classes can generally be identified: cationic, amphipathic, and hydrophobic. Data from publications and patents, excluding mutants of the same peptides, indicate that the majority of CPPs carry a net-positive charge. Amphipathic CPPs, which encompass both cationic and anionic peptides, constitute the largest class, while hydrophobic peptides represent only about 15% of the total [23,32]. This classification correlates with their interactions with cell membranes and other organelles [10]. Under physiological conditions, a significant percentage of peptides in all three CPP classes are positively charged.

Within the group of cationic CPPs (cCPPs), peptides are classified based on specific characteristics: they contain a continuous stretch of basic amino acids and lack the formation of an amphipathic helix in their 3D structure. Amphipathic CPPs (aCPPs) are chimeric peptides, with the most common structural motif being an amphipathic α-helix. Amphipathic α-helical CPPs possess a highly hydrophobic patch on one side, while the other side can be cationic, anionic, or polar. Hydrophobic CPPs (hCPPs) consist solely of non-polar residues or have very few charged amino acids, typically cationic, which are often derived from signal peptide sequences. In recent years, the discovery and characterization of novel natural peptide sequences with cell-penetrating activity have expanded the length limits and modified the physicochemical properties of constituent residues, enabling the design of neutral and even anionic CPPs.

### 2.1. Cationic CPPs (cCPPs)

Cationic cell-penetrating peptides (cCPPs) are a subclass of CPPs defined by a high density of basic amino acids, predominantly arginine (Arg) and lysine (Lys), which confer a net positive charge under physiological conditions. Arginine, due to its guanidinium side chain, exhibits unique hydrogen-bonding capabilities, forming stable bidentate interactions with anions such as phosphates and sulfates on the cell surface. These interactions enhance the cellular uptake efficiency by facilitating electrostatic binding to negatively charged glycosaminoglycans, including heparan sulfate. In contrast, lysine, with its primary amine side chain, shows lower affinity for these anions, leading to reduced internalization compared to arginine-rich sequences. For instance, polyarginines (e.g., R8–R10) demonstrate significantly higher translocation efficiency than polylysines of equivalent chain length, highlighting the superior electrostatic and hydrogen-bonding properties of the guanidinium group. Unlike amphipathic CPPs, cCPPs generally lack the formation of an amphipathic helix in their three-dimensional structure, typically adopting disordered or random coil conformations. This structural feature, combined with their cationic nature, enables cellular entry through mechanisms such as direct translocation and endocytosis. Prototypical examples of cCPPs include the HIV-1 Tat peptide (49–57) and polyarginines, which are widely used for cargo delivery in various biological contexts [33,34,35].

#### 2.1.1. Tat

A cCPP contains a continuous stretch of basic amino acids, and its 3D structure does not form an amphipathic helix. The first discovered cCPP was derived from the HIV-1 protein Tat, a potent trans-activator essential for virus replication, which is considered a prototypic cCPP [36]. The HIV-1 Tat-protein-derived Tat peptide, denoted as Tat (residues 49 RKKRRQRRR 57, functional region III [3]), is fully polymorphic and unable to adopt any specific secondary structure, regardless of its environment. Tat remains disordered in the presence of phospholipids [37,38], exhibiting a random coil conformation in solution [39]. In aqueous solutions, polyarginine peptides typically form unstructured or polyproline type II helical conformations. This phenomenon is likely due to the strong repulsive interactions between the guanidinium groups of the arginine residues [40,41,42,43]. The abundant arginine residues in Tat, along with the guanidinium moieties in this peptide, facilitate strong binding to carboxylic, sulfate, and phosphate groups on the surface of cell membranes. These unique binding properties induce endocytosis and/or membrane transduction, enabling the transport of Tat and its cargo across the blood–brain barrier and cellular membranes [44]. Tat is known to aggregate at phospholipid membranes and occasionally fuse vesicles [45,46,47]. Previous studies have shown that the deletion of one arginine residue from either the amine terminus (Tat50–57) or the carboxyl terminus (Tat49–56) resulted in a significant (80%) loss in intracellular fluorescence compared to the parent sequence (Tat49–57) [48]. Tat and its derivatives have been evaluated as potential carriers of molecules for intracellular release in the treatment of various diseases [49,50,51,52,53,54], as well as possible bactericides against Gram-positive and Gram-negative bacteria. Additionally, there are studies reporting the antifungal activity of Tat [55,56,57]. Recently, significant progress has been made in utilizing Tat complexes, which have greatly contributed to the advancement of therapeutic approaches based on this CPP. Numerous Tat-conjugated drugs were under clinical development for applications as diverse as heart diseases (NCT00785954, phase II, completed 2011), intraocular inflammation (NCT02235272, phase III, completed 2016; NCT02508337, completed 2017, phase III), and acute inner ear and hearing loss (NCT02561091, completed 2017, phase III) [58,59,60] (see Table 1).

#### 2.1.2. PTD4

Several modified PTD peptides exhibit significantly enhanced cell penetration capabilities compared to the original TAT PTD, offering potential for the development of novel therapeutic strategies [61]. One such modified peptide, known as PTD4 (a less basic Ala-scan analog of the TAT peptide, YARAAARQARA-NH2), has garnered significant attention due to its improved transduction efficiency. PTD4 contains fewer arginine residues than the original TAT(49–57) sequence, yet it demonstrates superior cell membrane translocation efficiency [62]. This enhanced efficiency is attributed to an optimized balance between positive charge and hydrophobicity, which facilitates more effective interactions with negatively charged cell membranes. Consequently, PTD4 promotes entry into the cell interior via mechanisms involving outer membrane receptors or transmembrane channels, facilitating efficient intracellular delivery of cargo [63].

Preclinical investigations have extensively explored the potential of PTD4 for drug delivery applications. Initial studies showed that PTD4 could efficiently transport therapeutic peptides and proteins across cellular membranes, enhancing their intracellular availability. In vitro experiments using cell lines demonstrated that PTD4-conjugated cargoes exhibited significantly higher uptake compared to unmodified TAT PTD [61].

Further in vitro studies using mouse B16-F1 and human A875 and SK-MEL-5 melanoma cells, along with in vivo studies in a B16-F1 melanoma mouse model, demonstrated that the PTD4-mediated delivery of chemotherapeutics like dacarbazine enhances tumor regression while reducing systemic toxicity. Combined PTD4–apoptin/dacarbazine treatment, with a 50% reduction in dacarbazine dosage, achieved similar antitumor efficacy without the hematologic side effects typically associated with full-dose dacarbazine. These findings underscore the potential of PTD4 to enhance chemotherapeutic efficacy, enabling dose reduction, minimizing adverse effects, and ultimately improving patient outcomes [64]. In another study, apoptin was fused with PTD4 to facilitate its delivery across cell membranes. The PTD4–apoptin complex induced apoptosis in cervical carcinoma cells by increasing active caspase-3 levels. Interestingly, apoptin-induced Mfn-2 accumulation was unaffected by Bcl-2, while Bcl-2 inhibited apoptin-mediated AKT activation. In vivo, cervical carcinoma xenografts treated with PTD4–apoptin for seven days exhibited significant tumor reduction compared to PTD4-GFP controls. TUNEL analysis confirmed apoptosis induction by PTD4–apoptin, highlighting its potential as an anticancer agent in cervical carcinoma [65].

On another front, Wang et al. designed anticancer agents targeting CyclinD1/CDK4 and CyclinD3/CDK4 complexes, which are key regulators of cell cycle progression. They synthesized chimeric peptides derived from key motifs in these complexes and conjugated them to PTD4. The resulting peptides—PTD4-D1, PTD4-D3, and PTD4-K4—exhibited significant antiproliferative effects by competing with CyclinD/CDK4 complexes, leading to G1/S phase arrest and apoptosis. Tumor challenge experiments further demonstrated potent antitumor activity with minimal side effects, positioning these PTD4-conjugated peptides as promising lead compounds for cancer therapy [66].

PTD4 has also been applied to inhibit the expression of connective tissue growth factor (CTGF), thereby reducing fibrosis and minimizing scar formation [67]. A notable example is the AZX100-PTD4 conjugate, which comprises a phosphorylated peptide analog of HSP20 (WLRRAS(phospho)APLPGLK) covalently conjugated to PTD4 [68]. Preclinical studies have shown that AZX100 effectively reduces stress fiber formation, induces morphological changes in human dermal keloid fibroblasts, and minimizes scar tissue formation in vivo. As a result of these promising outcomes, AZX100-PTD4 has advanced to clinical testing, specifically targeting keloid scar reduction. To date, it has successfully completed three Phase II clinical trials, underscoring the therapeutic potential of PTD4 in treating human diseases characterized by excessive fibrosis and scarring (NCT00811577; NCT00825916; NCT00892723) (see Table 1).

The continued evaluation of PTD4 in clinical settings remains crucial for fully understanding its therapeutic potential. Future studies should focus on optimizing dosing regimens, improving delivery specificity, and minimizing potential immunogenic responses. Moreover, exploring the use of PTD4 for delivering a broader range of therapeutic agents, including nucleic acids, proteins, and small molecules, will expand its clinical applicability. The efficacy of PTD4 in clinical applications, particularly for conditions requiring intracellular delivery of therapeutic agents, is supported by multiple studies [63,68,69,70,71,72,73].

#### 2.1.3. Penetratin

Another widely studied cCPP is Penetratin (43 RQIKIWFQNRRMKWKK 58), which corresponds to the 16 residues of the third α-helix of the Antennapedia homeodomain of Drosophila. This region is responsible for the translocation of the entire homeodomain across cell membranes and is structurally conserved in a wide range of homeodomains, indicating high evolutionary conservation [74,75,76,77]. A minimal active translocation region has been identified through cell penetration assays using human cell cultures. These assays have revealed that the C-terminal segment 52 RRMKWKK 58 of the original sequence is necessary and sufficient for efficient membrane translocation, retaining 60% of the full-length Penetratin uptake efficiency [78]. Penetratin exhibits structural variations depending on its environment. Studies on its secondary structure when interacting with charged vesicles have demonstrated that it can adopt a helical conformation [79] or a dominant β-structure [80], depending on the specific conditions. Experiments using Penetratin variants in membrane-mimicking environments have indicated that the translocation process does not involve chiral recognition by a receptor, and induced helical secondary structure does not appear to be necessary [75,76]. The presence of positively charged basic amino acids contributes to its solubility in water, leading to a predominantly random coil conformation, although a significant contribution from β-structure is also observed [81]. While charged residues play a crucial role in the uptake of cationic CPPs, other residues are also important for membrane binding and translocation. The significance of aromatic residues in the cellular uptake of Penetratin has been demonstrated, with tryptophan 48 and 56, as well as phenylalanine 49, identified as crucial for efficient translocation. Substituting Trp48 with Phe has been shown to greatly impair cellular uptake. The authors suggest that the electrostatic interactions established by Penetratin on the cell surface stabilize it, and they propose that Trp48 and the positively charged amino acids promote the creation of inverted micelles, which capture the peptide and facilitate its transport to the cytoplasm [74,78,82,83]. These findings suggest a delicate balance between hydrophobic and positively charged residues that can impact the interaction with membranes and the translocation process [84]. The ability of Penetratin to translocate across cell membranes has established it as a widely utilized CPP for the development of drug delivery systems. It enables the direct delivery of diverse cargo types, including peptides, nanoparticles, proteins, and nucleic acids, into the cell [85,86,87,88,89,90]. The evidence supporting Penetratin’s cell-penetrating capability includes studies demonstrating its entry into various cell types such as murine fibrosarcoma cells (WC/1), chicken fibroblast cells (CEC-32), chicken monocytic cells (HD-11), human fibroblast cells (SV 80), and human monocytic cells (MonoMac-6) [91].

#### 2.1.4. Polyarginines

Oligoarginines or polyarginines have been reported to penetrate various types of cells efficiently without causing significant cytotoxicity, under non-invasive conditions and at lower concentrations (in the range of tens of micromolar). They have been used to facilitate the uptake of a variety of small molecules and biomolecules [92,93,94,95,96,97,98]. It is known that the hepta-L-arginine (R7) sequence represents the lowest threshold for cellular uptake, and increasing the number of arginines is positively correlated with the uptake level. For delivery purposes, the optimal range is between R8 and R10 [33,48,95,99]. Several studies suggest that the R8 peptide possesses favorable characteristics in terms of achieving a balance between membrane uptake and cytosolic release [35,100,101,102]. It is important to emphasize that cellular uptake is not solely determined by charge, chirality, or backbone length. It has been determined that the guanidine headgroup of arginine serves as the critical structural component responsible for the observed biological activity. This importance was confirmed by the inability of citrulline heptamers to enter cells [33,48,100]. The guanidinium headgroup (arginine) is capable of hydrogen donation, while the oxygen of urea (citrulline) acts as a hydrogen bond acceptor. This interaction is particularly evident in the strong ability of guanidine to form a stable bidentate hydrogen bond with anions like phosphate or sulfate [35]. The exact mechanism of the internalization of polyarginine peptides is currently the subject of ongoing research. Numerous investigations have identified multiple uptake mechanisms that seem to be influenced by factors such as peptide concentration, the transported cargo, and the length of the peptide chain. Among these mechanisms are clathrin-mediated endocytosis (CME) [103,104] and macropinocytosis [94,105,106].

##### ATX-101

ATX-101 (MDRWLVKWKKKRKIRRRRRRRRRRR) is a cationic CPP composed of three parts: an AlkB homologue 2 proliferating cell nuclear antigen (PCNA)-interacting motif (APIM; RWLVK), an SV40 nuclear localization signal (KKKRK), and the CPP undeca-arginine (R11) [107]. Through the APIM motif, PCNA interacts with numerous cellular proteins that are crucial for cellular stress responses, DNA damage repair, intracellular signaling, apoptosis, metabolism, and antitumor immunity. Consequently, ATX-101 enhances the efficacy of various anticancer drugs by disrupting PCNA/APIM interactions. This includes DNA-damaging agents, microtubule-targeting drugs, molecular targeting agents (such as p38 MAPK and EGFR inhibitors), and γ-irradiation [108,109]. Müller et al. demonstrated that ATX-101 induces caspase-dependent apoptosis in multiple myeloma cell lines and primary cancer cells, independent of the cell cycle phase. Additionally, ATX-101 increased the sensitivity of multiple myeloma cells to melphalan, a DNA-damaging agent commonly used in the treatment of multiple myeloma [107]. Preclinical studies demonstrated that ATX-101 rapidly penetrated cells and targeted PCNA/APIM-containing protein complexes, which are crucial for cell survival and DNA repair. A completed Phase I clinical trial of ATX-101 (NCT01462786) revealed a favorable safety profile, with no significant adverse effects reported. ATX-101 is currently being evaluated in several clinical trials (see Table 1). For instance, in a Phase I/II study involving patients with platinum-sensitive fallopian tube and primary peritoneal cancer, ATX-101 was combined with platinum-based chemotherapy (NCT04814875). Additionally, a Phase II study (NCT05116683) has been initiated to assess the efficacy of ATX-101 as a monotherapy in sarcoma, with the aim of evaluating its antitumor activity and further characterizing its safety and pharmacokinetics.

cCPPs have garnered significant interest for their potential in the intracellular delivery of various biomolecules, including nucleic acids, proteins, and small drugs. However, the efficiency of cCPPs in facilitating the release of these biomolecules into the cytoplasm or nucleus is often hindered by endocytic sequestration. Once internalized, many cCPPs are trapped within endosomes and subsequently directed towards lysosomal degradation, which limits their functional delivery [110,111].

##### (R-X-R)_4_, X = 6-Aminohexanoic Acid

To overcome this challenge, innovative CPPs have been engineered, such as spaced oligoarginine conjugates linked with 6-aminohexanoic acid ((R-X-R)_4_, X = 6-aminohexanoic acid). These novel CPPs have shown the ability to internalize vectors via endocytic pathways that involve proteoglycans, which are essential components of the cell surface and play a crucial role in endocytosis [112]. Unlike traditional cCPPs, the spaced oligoarginine conjugates can escape endosomal entrapment and avoid lysosomal degradation, thereby facilitating the delivery of their cargo to the nuclear compartment. This capability is particularly advantageous for applications requiring direct nuclear delivery, such as gene editing and nucleic acid-based therapies [113,114,115]. In HeLa pLuc705 cells, conjugates of branched and linear (R-X-R) peptides—with X being a 6-aminohexanoic acid—are primarily internalized via an energy-dependent, clathrin-mediated pathway, with additional contributions from caveolae-mediated endocytosis. However, advanced cellular techniques are required to delineate the predominant endocytic mechanisms specific to these peptide–cargo conjugates [110,111].

A phase II clinical study registered under NCT00451256 aimed to evaluate whether immersing the excised saphenous vein in a novel antisense anti-c-myc solution (AVI-5126) would prevent graft failure after one year, compared to immersion in physiological saline (placebo) before grafting (see Table 1). The study was discontinued because the authors determined that the predefined efficacy outcomes described in the protocol would not be achieved. Consequently, the safety and efficacy evaluation of AVI-5126 for use in vein grafts prior to cardiac bypass graft (CABG) surgery was not completed. Further studies will be necessary to determine if this pharmaceutical formulation could have potential use in the future.

In cancer surgery, the complete resection of tumor lesions is crucial for optimal outcomes. However, distinguishing between tumor and normal tissues is challenging, often leading to residual tumor tissue and subsequent recurrence [116]. Intraoperative imaging with fluorescent molecular probes aids surgeons in visualizing tumor lesions and their boundaries, enhancing precision and reducing the likelihood of residual disease [117]. CPPs are crucial in imaging applications, especially when conjugated with agents like fluorophores and radioisotopes. These conjugates improve the imaging of cells and tissues, greatly enhancing diagnostic precision and advancing research capabilities. Specifically, CPP conjugates with fluorescent probes have been successfully designed to precisely delineate tumor boundaries, facilitating surgical removal.

#### 2.1.5. AVB-620

AVB-620 is a protease-cleavable peptide conjugated with the fluorophores Cy5 and Cy7 at the cationic and anionic terminals, respectively. To improve water solubility, an α-aminoxyl-ω-methoxy PEG (mPEG, Mw ≈ 2000) moiety was conjugated. Its unique hairpin structure enables efficient fluorescence resonance energy transfer (FRET) between the fluorophores. The peptide linker is designed to be cleaved by MMP2 and MMP9, enzymes highly expressed in human breast cancer cells. MMPs hydrolyze AVB-620, causing it to be retained in the tissue and triggering a ratiometric fluorescence color change. This change can be visualized using camera systems that simultaneously capture fluorescence and white light images [117,118,119]. Consequently, surgeons can visualize and remove the tumor during surgery. In the phase I clinical trial (NCT02391194), AVB-620 was proven to be safe and effective for detecting tumor-positive tissue during surgery [120]. A phase II, single-arm, open-label study in women with primary breast cancer undergoing surgery evaluated how the timing of AVB-620 administration affects fluorescence and imaging accuracy, as well as its ability to differentiate between malignant and non-malignant tissues (NCT03113825). The intraoperative imaging of surgical specimens infused with AVB-620 enabled real-time tumor detection. The infusion of AVB-620 is safe and has the potential to enhance the intraoperative detection of malignant tissue during breast cancer surgeries.

#### 2.1.6. Polylysines

It is known that other cationic poly amino acids, such as polylysines (PL), enhance the uptake of small molecules, peptides, proteins, or even larger particles, like viruses, into cells. PL peptides have been used for the delivery of methotrexate [121,122,123], 5-Fluorouracil [124], oligonucleotides [125,126,127], albumin [128] or horseradish peroxidase [128,129], and adenovirus [130,131,132,133]. Polylysine peptides have been demonstrated to bind to the membrane surface via electrostatic interactions, subsequently inserting into the membrane interface region, facilitating their translocation [134]. Contrarily, polyarginines exhibit higher cellular uptake efficiency compared to lysine polymers with similar chain lengths. The distinction in membrane translocation between polyarginine and polylysine was observed in early studies on CPPs, where investigations comparing oligoarginine and polylysine revealed the distinctive properties of the guanidinium group in arginine concerning proteoglycan binding, encompassing both the affinity and clustering of binding [33,134,135].

##### TransMTS^®^

TransMTS^®^ proprietary peptides are simple, straight-chain peptides characterized by two distinct domains. The main core of the peptide consists of consecutive lysine residues, which carry a positive charge under physiological conditions. This positively charged core forms non-covalent (electrostatic) bonds with negatively charged macromolecules intended for transport. The second domain is a PTD, crucial for delivering the macromolecule to the target site. Each peptide is flanked by two identical PTDs at its termini, enhancing its ability to penetrate cell membranes and effectively transport the macromolecule into target cells. The positively charged lysine core not only facilitates binding to negatively charged macromolecules, such as nucleic acids or proteins, but also enhances the overall stability of the complex in biological environments. The electrostatic interactions ensure that the macromolecule is securely bound to the peptide, preventing premature release and degradation. The PTD domains, often derived from well-known transduction sequences like the TAT peptide from HIV-1, are responsible for the efficient cellular uptake of the peptide–macromolecule complex [136]. These domains exploit the cell’s natural endocytic pathways, allowing the complex to traverse cellular membranes and reach intracellular targets. By flanking the peptide with two PTD domains, the design maximizes transduction efficiency, offering a robust delivery mechanism [137].

Overall, the unique structure of TransMTS^®^ peptides, with their bifunctional domains, represents a sophisticated approach to intracellular delivery. This design not only improves the targeting and uptake of therapeutic macromolecules but also enhances their therapeutic potential by ensuring precise delivery to specific intracellular locations (see Table 1). A TransMTS^®^–botulinum toxin A (the active ingredient in Botox) conjugate is currently in Phase III clinical trials for cervical dystonia (involuntary and painful neck contractions) and plantar fasciitis (heel pain) (NCT03608397) [138]. Botulinum toxin A conjugates have been developed for various therapeutic and cosmetic applications, including the treatment of moderate to severe lateral canthal lines (NCT02580370) and primary axillary hyperhidrosis (NCT02565732) [139,140,141].

#### 2.1.7. Z12

Numerous studies have explored the utility of the Epstein–Barr virus transactivator protein ZEBRA as a vector for the delivery of diverse cargo proteins. Investigations focusing on truncated versions of ZEBRA have identified a minimal domain (MD), comprising amino acids 170-220, essential for its internalization capabilities [142]. Within this domain, a specific peptide segment termed *Z12* has been demonstrated to efficiently transport reporter proteins across various normal and tumor cell lines. Notably, the internalized cargo proteins retained their functionality, and no cellular toxicity associated with MD was observed. The mechanism of MD translocation across the cell membrane involves binding to heparan sulfate proteoglycans (HSPGs) on the cell surface, occurring primarily through direct translocation across the lipid bilayer rather than via endocytosis [143].

The peptide *Z12* (KRYKNRVASRKCRAKFKQLLQHYREVAAAKSSENDRLRLLLK) has been employed in the development of cellular-based vaccines by conjugating its sequence with multi-epitopic antigens. Treatments with Z12-formulated vaccines have extended survival rates in three tumor models, with the most significant increase observed in an aggressive brain cancer model. Analysis of tumor sites showed favorable immune modulation, indicating that these vaccines not only promote an integrated immune response but enhance the persistence and homing of CD8+ effector T cells to the tumor site. Furthermore, the presence of Th1 polarized CD4+ T cells was noted, contributing to potent antitumor immunity in various models, including gliomas [144]. These findings underscore the versatility and effectiveness of Z12 as a carrier for multi-epitope antigens, showcasing its potential in advancing cancer immunotherapies. The ability of Z12 to promote persistent and targeted immune responses highlights its value as a tool for developing innovative therapeutic strategies against cancer [145,146]. Furthermore, this promising potential has led to the initiation of a Phase I clinical trial (NCT04046445) to evaluate the safety, tolerability, and antitumor activity of ATP128, VSV-GP128, and BI 754091 in patients with stage IV colorectal cancer. This trial represents a critical step in translating the preclinical successes of Z12 into clinical applications in humans and animals [147].

Several studies have investigated CPPs derived from the ZEBRA protein, beyond the Z12 fragment, in the development of therapeutic cancer vaccines. These investigations have evaluated various truncated forms of ZEBRA for their structural properties, in vitro transduction efficiency, and their ability to induce CD4 and CD8 T cell responses in vivo. The findings underscore the critical role of selecting appropriate CPP sequences and adjuvants to optimize antitumor immunity. Optimal CPP-adjuvant combinations significantly enhance immune responses and control tumor growth in aggressive tumor models, demonstrating the promising potential of ZEBRA-derived CPP-based vaccines in cancer therapy [145,148,149].

### 2.2. Amphipathic CPPs (aCPPs)

Multiple investigations have indicated that among the identified CPPs to date, amphipathic peptides are the most abundant, accounting for approximately 40% of them. Amphiphilic CPPs (aCPPs) consist of both polar and non-polar amino acid regions, with the non-polar regions being rich in hydrophobic amino acids like Ala, Val, Leu, and Ile. The amphipathic α-helix is the most common structural motif of many amphipathic CPPs. Amphipathic α-helical CPPs exhibit a highly hydrophobic region on one side, while the other side can be cationic, anionic, or polar. Previous studies have demonstrated that variations in conditions can lead to different secondary structures in the same sequence, thereby altering its capacity to interact with the hydrophobic/hydrophilic interface [39]. For example, the α-helical MAP (model amphipathic peptide) spontaneously inserts into the lipid monolayer, exhibiting strong interactions with negatively charged phospholipids. Conversely, the structural analysis of peptide/lipid interactions of MPG (N-methylpurine DNA Glycosylase) revealed that its β-sheet structures are more responsive to external forces compared to its α-helical structures [150].

Based on their sequence, length, and lipid association, amphipathic CPPs can be classified into primary amphipathic, secondary amphipathic, β-sheet amphipathic, and proline-rich categories [151,152].

#### 2.2.1. Primary Amphipathic CPPs (paCPPs)

Primary amphipathic CPPs (paCPPs) can be defined as the sequential arrangement of a hydrophobic residue domain with a hydrophilic residue domain. Numerous paCPPs are chimeric peptides, with several being derived from partially covalently linking a hydrophobic domain to a nuclear localization sequence (NLS). paCPPs generally exhibit a longer length compared to cationic CPPs, typically consisting of 20 or more amino acids, which allows them to traverse the hydrophobic core of membranes. Many paCPPs demonstrate a strong affinity for both neutral and anionic membranes, potentially leading to lipid reorganization and disruptions in membrane structure. While the majority of paCPPs are chimeric or synthetic in nature, certain sequences are derived from proteins. Examples include the VE-cadherin-derived CPP, pVEC [153], the N-terminal sequence (1–30) of the bovine prion protein, BPrPp(1–30), the N-terminal sequence (1–28) of the mouse prion protein, MPrPp(1–28) [154,155], and the N-terminal sequence (1–22) of the tumor suppressor p14ARF, ARF(1–22) [7].

##### pVEC

The uptake of pVEC (LLIILRRRIRKQAHAHSK) occurs through a non-endocytic translocation mechanism without causing changes in plasma membrane permeability or cell morphology. Once internalized, it predominantly localizes to nuclear structures, making it a potent carrier for peptide nucleic acids (PNAs) and proteins [153]. Studies investigating the pVEC sequence to determine the significance of each residue and the impact of single substitutions on translocation ability have demonstrated the crucial role of the N-terminal hydrophobic region of pVEC in efficient cellular translocation [156]. pVEC has also been found to enhance the translocation of homing peptides that selectively target molecular markers on tumor cells. One such homing peptide is the cyclic peptide PEGA (CPGPEGAGC), which has previously shown accumulation in breast tumor tissue in mice. In vitro studies have shown that PEGA peptide conjugated with the CPP pVEC can be taken up by various breast cancer cells. Additionally, the conjugation of the anticancer drug chlorambucil to pVEC-PEGA has been demonstrated to significantly increase drug efficacy by more than fourfold, leading to a reduction in the clonogenic survival of MCF-7 cells, thus highlighting its potential as a carrier for anticancer drugs [157,158,159].

##### bPrPp(1–30)

A notable paCPP is the bPrPp(1–30) peptide (MVKSKIGSWILVLFVAMWSDVGLCKKRPKP). Its structure and membrane interaction have been examined using spectroscopy techniques. Circular dichroism (CD) spectroscopy demonstrated that the peptide adopts predominantly an α-helical conformation in zwitterionic bicelles and DHPC micelles, while exhibiting a lesser degree of α-helical structure in partially charged bicelles. The structure of bPrPp(1–30) was determined in DHPC micelles, revealing an α-helix spanning residues Ser8 to Ile21. The peptide induced some degree of ordering within the bilayer, consistent with positive hydrophobic mismatch, and its stable helical conformation allowed insertion at a transmembrane position within the bilayer [160]. The internalization of bPrPp predominantly occurs through macropinocytosis, a fluid-phase endocytic process initiated by binding to cell-surface proteoglycans [155].

##### MPrPp(1–28)

Another N-terminal sequence, MPrPp(1–28) (MANLGYWLLALFVTMWTDVGLCKKRPKP), exhibits a strong propensity for aggregation and β-structure formation, particularly in its interaction with negatively charged phospholipid membranes. It has been observed that the conformational characteristics adopted by the MPrPp(1–28) peptide vary significantly depending on the environment. In the presence of neutral POPC or negatively charged POPG vesicles, the CD spectra of PrP(1–28) indicate a predominant α-helical or β-structure, respectively [154]. The PrPp(1–28) segment may play a crucial role in the cellular transport of prion proteins and their infectivity. This is attributed to the potent β-structure induction within the (1–28) region facilitated by the interaction with a negatively charged membrane surface. Notably, MPrPp(1–28) is capable of transporting large hydrophilic cargoes across the cell membrane, likely through an α-to-β transition catalyzed by a negatively charged lipid surface.

##### ARF(1–22)

Johansson et al. conducted a study to assess the impact of the ARF(1–22) peptide (MVRRFLVTLRIRRACGPPRVRV) on cell proliferation, apoptosis induction, stability, and cellular uptake mechanisms. ARF (1–22) demonstrated a dose-dependent decrease in cell proliferation and induced apoptosis. The evaluation of peptide stability revealed that ARF (1–22) remained stable within cells for a minimum of three hours, with consistent concentrations of both intact and degraded peptide over time. This suggests that uptake and degradation occur at similar rates during the initial hours. The primary uptake mechanism for ARF (1–22) was identified as vesicular uptake, indicating endocytosis as the main pathway. This uptake mechanism enables the peptide to transport bioactive charges to the nucleoli of cells. For instance, when ARF (1–22) was conjugated with splice-correcting PNA, correct splicing was restored [7].

##### MPG and Pep-1

Two other relevant paCPPs are N-methylpurine DNA Glycosylase (MPG, GALFLGFLGAAGSTMGAWSQPKKKRKV) and Pep-1 (KETWWETWWTEWSQPKKKRKV). Both peptides consist of three domains: firstly, a variable N-terminal hydrophobic motif; secondly, a hydrophilic lysine-rich domain derived from the NLS (nuclear localization sequence) of SV40 (simian virus 40) large T-antigen (KKKRKV); and thirdly, a linker domain (WSQP) containing a proline residue. This linker domain improves the flexibility and integrity of both the hydrophobic and hydrophilic domains [161,162,163,164,165]. However, the peptides mainly differ in their hydrophobic domain. The MPG hydrophobic domain (GALFLGFLGAAGSTMGA) is derived from the fusion sequence of the HIV glycoprotein 41 and is necessary for efficient targeting to the cell membrane and cellular uptake. On the other hand, the Pep-1 hydrophobic motif (KETWWETWWTEW) originates from a tryptophan-rich cluster, which is also crucial for efficient targeting to the cell membrane and for forming hydrophobic interactions with proteins [165]. Additionally, a cysteamine group is present at the C-terminal, and an acetyl group caps the N-terminus. Under oxidizing conditions, dimers may form due to the disulfide linkage of cysteamine groups [166].

MPG was originally designed for the rapid and efficient delivery of plasmid DNA into the nucleus [161]. Variations and optimizations were subsequently made for siRNA delivery [167,168,169,170]. Subsequently, through a single mutation substituting the second Lys residue in NLS with Ser (changing KKKRKV to KSKRKV, denoted as ΔNLS), the nuclear translocation function was eliminated, allowing for the swift release of siRNA in the cytoplasm. This modification enabled the effective down-regulation of target mRNA [163,171]. Research findings have demonstrated that MPGΔNLS exhibits a robust interaction with the phospholipid cell membrane by means of its hydrophobic gp41 sequence. This sequence has the ability to transiently adopt a β-sheet structure and integrate into the plasma membrane. Consequently, the insertion of the β-sheet brings about a temporary alteration in the organization of the cell membrane, generating a transient channel. This channel facilitates the entry of the siRNA/MPGΔNLS complex into the cytoplasm [172]. The cationic end plays a major role in siRNA condensation, while the hydrophobic region stabilizes the CPP/siRNA complex via intermolecular hydrophobic interactions. MPG has been demonstrated to interact with the extracellular matrix via negatively charged HSPGs. The initiation of cellular uptake is a highly dynamic mechanism in which the binding of MPG to the extracellular matrix rapidly triggers a remodeling of the actin network, involving the activation of the GTPase Rac1 [173]. In another study, MPG has been utilized to enhance antigen cross-presentation and elicit an antitumor immune response. When MPG binds to antigens encapsulated in nanovaccines, it influences the spatial and temporal intracellular localization of antigens, promoting antigen cross-presentation and stimulating antigen-specific immune responses, particularly cytotoxic T lymphocyte (CTL) responses. This innovative approach has demonstrated promising results in enhancing the efficacy of nanovaccines for cancer immunotherapy [174]. Experimental evidence suggests that most CPPs are taken up via endocytosis, although MPG and Pep-1 uptake are known to be energy independent [162,163].

Pep-1 undergoes a conformational rearrangement upon interaction with lipids, and its insertion into the membrane is accompanied by segregation of lipids, membrane disorganization, and transient pore formation, allowing for transient ionic currents [175,176]. Evidence indicates that the highly charged hydrophilic domain is responsible for the first contact with the membrane due to electrostatic interactions between the polar head group of phospholipids and the positive charges of Pep-1, and that the dehydration and insertion of the hydrophobic domains promote membrane destabilization [166,177,178,179]. It has been observed that vesicles mimicking normal and cancer cell membranes exhibit differential responses to the CPP Pep-1. Pep-1 exhibits selectivity towards model membranes based on their composition, binding sites, and peptide concentration. The interaction of Pep-1 with cancer cells is relatively stronger compared to normal cells in terms of the C=O group, whereas the interaction of Pep-1 with normal cells is relatively stronger compared to cancer cells in terms of the phosphate group [180]. Hydrophobic interactions have been shown to be the primary driving force behind peptide interactions with normal cell membranes, whereas electrostatic interactions play a dominant role in peptide interactions with cancer cell membranes [181]. Cell selectivity is primarily determined by an elevation in the concentration of acidic components on the cancer cell wall rather than a slight increase in the level of phosphatidylserine (PS) on the outer surface of the cancer cell membranes [182]. Numerous studies have shown that the presence of the membrane induces various secondary structures in Pep-1. However, it remains unclear which structure is crucial for transmembrane administration [183,184]. Pep-1 has the capability to transport a wide range of peptides and proteins into various cell lines, regardless of their nature and size, while preserving their biological activity. These cell lines include neuronal cells [185], pancreatic cells [186,187], neural retinal cells [188], macrophages [189], and hepatocytes [190]. The mechanism by which Pep-1 delivers active macromolecules has been determined to be independent of the endosomal pathway. Furthermore, the dissociation of the Pep-1 particle–macromolecule complex occurs immediately upon crossing the cell membrane [191]. Pep-1 offers several advantages, including high nanomolar affinity for most proteins and peptides, stability in physiological buffers, and the absence of toxicity. These attributes make it a promising candidate for the development of therapeutic applications utilizing covalent protein transduction domains.

##### PEP-010

PEP-010 is a CPP specifically designed to disrupt key intracellular interactions involved in apoptosis, particularly those between caspase-9 and protein phosphatase 2A (PP2A). While the exact sequence of PEP-010 is proprietary and not disclosed in clinical study summaries for intellectual property and confidentiality reasons, it is known to be a bifunctional peptide comprising 30 amino acids. It includes the DPT sequence, which facilitates cell penetration, and the Pep-1 sequence, which actively interferes with PP2A [192]. Upon administration, the cell-penetrating segment of PEP-010 facilitates its entry into the cytosol, where the interfering segment disrupts the interaction between caspase-9 and PP2A. This disruption leads to the release and activation of caspase-9, thereby restoring apoptosis in tumor cells [193,194]. Activated caspase-9 induces caspase-dependent apoptosis. PP2A, a serine/threonine phosphatase, plays a crucial role in regulating cell growth and DNA damage repair.

As a bifunctional peptide, PEP-010 can penetrate cells and interfere with essential protein interactions, thereby promoting apoptosis in cancer cells. This mechanism of action has been validated through both in vitro and in vivo studies, showing efficacy in patient-derived xenograft (PDX) models of triple-negative breast cancer (TNBC) and hormone receptor-positive, HER2-negative breast adenocarcinoma [195]. NCT04733027 is a clinical trial designed to test PEP-010 in humans for the first time. This Phase I trial aims to determine the safety, tolerability, and optimal dosage of PEP-010 when administered alone and in combination with other chemotherapy agents, specifically, paclitaxel and gemcitabine. The study seeks to collect preliminary data on the pharmacodynamics, pharmacokinetics, and potential therapeutic benefits of PEP-010 in treating cancer.

#### 2.2.2. Secondary Amphipathic CPPs (saCPPs)

The α-helical secondary structure of secondary amphipathic CPPs (saCPPs) is crucial for their interaction with biological membranes. However, their internalization efficiency depends on various properties, including charge, guanidinium content, and primarily amphipathicity, which interact with one another. saCPPs are generally shorter than paCPPs and form stable associations exclusively with negatively charged membranes. Although saCPPs can adopt a helical amphipathic structure, they do not deeply insert into the membrane, resulting in fewer changes to biological membranes compared to paCPPs. Typically, secondary amphipathic saCPPs do not possess a well-defined structure when in solution. They acquire their amphipathic properties through a change in secondary structure, which occurs when they interact with negatively charged polyanions, membranes, or glycosaminoglycans.

##### MAP

Numerous studies have demonstrated that amphiphilic saCPPs, like the model amphipathic peptide (MAP) (KLALKLALKALKAALKLA), an artificial CPP incorporating hydrophobic and hydrophilic residues on opposite sides of its helical structure, exhibit robust interactions with negatively charged phospholipids and readily insert into the lipid monolayer, thanks to its well-defined amphipathic α-helix. Studies on the cellular uptake kinetics of various CPPs have revealed that MAP exhibits faster uptake and has the ability to induce membrane leakage by altering membrane integrity, even at concentrations as low as 1 μM. Additionally, MAP has been found to exert a strong toxic effect on various cell lines [196,197]. For example, a recent study investigated whether coupling the drug tacrine with the MAP peptide could enhance the antiproliferative properties of the drug. Both MAP and its tacrine conjugate were found to exhibit high toxicity against two cancer cell lines, namely, breast (MCF-7) and neuroblastoma (SH-SY5Y) cells. Since the unconjugated MAP peptide exhibited the same cytotoxic effect, it can be inferred that the peptide functions more as a cell-killing agent rather than a CPP. This highlights the potential utility of MAP as an antimicrobial peptide (AMP) capable of rapidly destroying cell membranes, even at low concentrations (1 μM) [198]. Regarding MAP analogs with inverse charge, like MAP17 (QLALQLALQALQAALQLA), the evidence suggests that their membrane translocation ability stems from their amphiphilic nature [199].

##### M918

The saCPP class encompasses other peptides, such as M918 (MVTVLFRRLRIRRACGPPRVRV), a highly hydrophobic and positively charged peptide derived from the tumor suppressor protein p14ARF. This peptide, comprising amino acids 1–22 with positions 3–8 inverted, demonstrates efficient translocation into various cells without toxicity. M918 can be utilized either as a covalent conjugate or in a non-covalent complex with the cargo. Its internalization is independent of GAGs on the cell surface and primarily relies on macropinocytosis for cellular uptake. Consequently, M918 is an effective carrier for large cargoes, such as proteins and peptide nucleic acids (PNAs) [200,201].

##### GALA and KALA

The saCPP class includes anionic peptides, one of which is GALA (WEAALAEALAEALAEHLAEALAEALEALAA). GALA is a synthetic peptide composed of 30 amino acids, featuring a repetitive sequence of glutamic acid–alanine–leucine–alanine (EALA). The glutamic acids (E) create a pH-dependent negatively charged side chain. Additionally, GALA contains histidine and tryptophan residues that act as spectroscopic probes. The EALA motif allows the peptide to have a hydrophobic surface, enabling interaction with the bilayer when it adopts an α-helical conformation. GALA is soluble in water at neutral pH, but at an acidic pH, it undergoes a conformational change from a random coil structure to an amphipathic α-helix. When GALA binds to bilayer membranes, it assembles into a transmembrane peptide pore composed of approximately 10 helical monomers aligned perpendicular to the membrane plane [202]. Studies suggest that GALA incorporates into the vesicular bilayer and aggregates to form a transbilayer pore consisting of around 10 peptides (±2). It has been observed that the lipid composition of the bilayer model influences the peptide’s orientation and insertion mechanism [203]. Due to its net negative charges, GALA is commonly used in combination with other DNA or oligodeoxynucleotides (ODN) condensation reagents to create particles for effective gene transfection. By partially replacing glutamic acid with lysine in the GALA sequence, the KALA peptide is derived.

KALA (WEAKLAKALAKALAKHLAKALAKALKACEA) is a cationic, endosomolytic, and fusogenic peptide capable of binding to DNA, destabilizing membranes, and mediating DNA transfection. At physiological pH (7.4), KALA adopts an α-helical conformation and exhibits a pH-dependent ability to form pores, distinct from those formed by GALA. As a cationic peptide, KALA disrupts membranes, condenses DNA, and can deliver ODN and plasmid DNA into the cell nucleus without requiring additional condensation reagents. A conformational change from an α-helix to a random coil occurs as the pH shifts from neutral to acidic [202]. Studies on gene expression have shown that KALA significantly enhances gene expression in substrate-mediated transfections [204,205]. The optimization of this peptide has led to the development of an amphiphilic cationic system that serves as an efficient non-viral gene delivery vector, exhibiting improved DNA condensation ability and reduced cytotoxicity [206].

##### p28

p28 is a naturally occurring bacterial peptide that has recently attracted considerable attention as both an efficient CPP and a promising anticancer agent. p28 (Leu50-Asp77) is an amphipathic, α-helical peptide composed of 28 amino acids, derived from azurin—a 128-amino acid (14 kDa) copper-binding member of the cupredoxin family of redox proteins, secreted as a periplasmic protein by *Pseudomonas aeruginosa* [207]. As a post-translational, multi-target anticancer agent, p28 demonstrates the ability to penetrate a wide range of solid tumor cells [208]. Mechanistically, p28 exerts its effects through two primary pathways. After cellular internalization, p28 binds to both wild-type and mutant p53 proteins, inhibiting their ubiquitination and subsequent proteasomal degradation mediated by the constitutive photomorphogenesis 1 (Cop1) protein. This inhibition results in elevated levels of p53, inducing G2/M phase cell-cycle arrest and apoptosis, thereby contributing to tumor cell reduction and death [209]. Preclinical studies and early-phase clinical trials have demonstrated the safety and potential efficacy of p28 in treating various cancers (see Table 1), including solid tumors (NCT00914914), glioblastoma and central nervous system tumors (NCT06102525; NCT01975116), and hepatocellular carcinoma (NCT05359861). The dual role of p28 as a CPP and anticancer agent positions it as a unique and promising candidate in therapeutic development [210,211,212].

#### 2.2.3. Amphipathic β-Sheet Peptides

An amphipathic β-sheet peptide is characterized by the presence of both hydrophobic and hydrophilic regions, which are exposed to the solvent. When folded, this peptide exhibits amphipathicity. The folding process is influenced by various weak non-covalent interactions, including electrostatic interactions, hydrogen bonding, the hydrophobic effect, and van der Waals forces. It is their ability to form β-sheets that enhances their efficiency in penetrating cells [213,214]. Amphipathic peptides have a strong tendency to spontaneously form one-dimensional fibril structures that resemble amyloids, which subsequently self-assemble into higher-order fibrils. Despite the absence of sequence homology among constituent peptides, amyloid assemblies derived from different peptides exhibit many shared structural characteristics. Amyloid fibrils adopt a quaternary structure known as the “cross-β” structure. Each individual peptide assumes an extended β-strand conformation, with the amino acid side chains oriented perpendicular to the amide backbone. The β-strands self-associate to form β-sheets, which are stabilized by hydrogen bonding between neighboring amide backbone groups, as well as hydrophobic, aromatic, and Coulombic interactions between adjacent side chain groups. Cross-β architectures emerge when multiple β-sheets laminate together to form fibrils [213,214]. These self-assembled peptide biomaterials form a unique scaffold in the shape of a fibrillar network. This scaffold has diverse applications, including regenerative medicine and tissue engineering, particularly through the use of hydrogels made from various β-sheet peptides [215,216].

##### EAK16 and RAD16

Significant progress in the field of amphipathic self-assembling peptides has unveiled a wide range of sequences, one of which is EAK16 (AEAEAKAKAEAEAKAK). This specific sequence demonstrates ionic self-complementarity and consists of an equal number of cationic Lys (K) and anionic Glu (E) residues, alternating with hydrophobic Ala (A) residues. Through the optimization of the EAK16 peptide, several other peptides with immense potential as vaccine adjuvants or hydrogels for cell culture support have been developed. By substituting Lys with Arg (R) and Glu with Asp (D) in EAK16, a distinct self-assembling peptide named RAD16 or RADA16 is formed [217,218]. Similar to EAK16, RAD16 exhibits the ability to spontaneously generate stable hydrogels in physiological buffers. These remarkable properties, including favorable cell attachment, high stability, and biocompatibility, have led to the successful commercialization of RAD16 as PuraMatrixTM peptide hydrogels (AcN-(RADA)_4_-CONH_2_) [219].

##### MAX1, MAX8 and Q11

In the field of biomedical applications, significant progress has been made by replicating the molecular design features of EAK16 to create self-assembling β-sheet peptides. Two notable examples of these peptides are MAX1 and Q11, which possess the remarkable ability to spontaneously form β-sheet structures. This unique property makes them highly appealing for various biomedical applications, including drug delivery and tissue engineering. MAX1 is composed of alternating hydrophobic Val residues and hydrophilic, cationic Lys residues within the β-strands. These β-strands are interconnected by the turn sequence VdPPT, where V represents Val, DP represents D-proline, P represents proline, and T represents threonine [220]. MAX1 exhibits the remarkable ability to form viscoelastic hydrogels, which vary in stiffness based on temperature and salt concentration [221]. MAX8, one of its derivatives, displays rapid gelation kinetics, enabling the homogeneous encapsulation of mesenchymal stem cells and hepatocytes. Additionally, MAX8 ensures high cell survival rates after immobilization in the gel matrix [222]. In contrast, Q11 (QQKFQFQFEQQ) assembles into a highly intricate gel-like network of β-sheet peptides, serving as an optimal modular biomaterial platform. Studies have revealed that incorporating cell-adhesive ligands through the co-assembly of Q11 and N-terminally modified Q11 with RGD or IKVAV significantly enhances cell attachment, proliferation, and growth [215]. On the other hand, Q11 acts as an adjuvant when fused to a peptide antigen. By attaching epitopes to the N-terminus of Q11, the epitopes can be displayed on the surface of the assembled nanofibers without affecting the peptide assembly. The unmodified Q11 peptide does not generate an immune response. However, when Q11 is conjugated with different epitopes, it induces detectable immune responses that can persist for weeks. These Q11 conjugated fibers elicit strong antibody responses, highlighting the potential of Q11-based vaccines for providing long-term protection [223,224,225].

##### E1Y9

E1Y9 peptide (EYEYKYEYKY) is an emerging biomaterial with great potential for cell culture applications. It has demonstrated the ability to form hydrogels that promote cell growth and proliferation. Numerous studies have showcased the biocompatibility of E1Y9 hydrogels with various cell types, including 3T3-L1 cells and PC12 cells [216,226]. Furthermore, these hydrogels have exhibited low toxicity levels comparable to commercially available microtiter plate surfaces. These findings position E1Y9 hydrogels as a compelling and versatile candidate for a wide range of cell culture applications [227].

Amphipathic β-sheet peptides offer a promising alternative to α-helical amphipathic peptides as vectors for delivering drugs, proteins, and oligonucleotides into mammalian cells. Further investigations are currently being conducted to better understand the underlying mechanism and explore the potential of utilizing β-sheet peptides as transport vectors for biomolecules.

#### 2.2.4. Proline-Rich Amphipathic Peptides

Proline-rich peptides are a chemically and structurally diverse family of CPPs characterized by the presence of pyrrolidine rings from proline residues. These peptides tend to assume helical conformations responsible for their internalization and enter cells via caveolae- or lipid raft-mediated endocytosis. The cellular uptake of proline-rich peptides occurs through their binding to anionic phosphate groups followed by interaction with the lipophilic region of the cell membrane. Proline-rich peptides are particularly effective, demonstrating efficient cellular uptake and low cytotoxicity [228]. Examples of these peptides include SAP (sweet arrow peptide) and its derivatives [229,230,231], bactenecin-7 (Bac-7) and its derivatives [232], and polyprolines [233].

##### SAP

SAP is an intracellular delivery peptide ((VRLPPP)3) derived from the natural sequence of the N-terminal domain of γ-zein VHL(PPP)8 [234]. SAP adopts a polyproline II helical structure (PPII) in an aqueous buffer, is highly soluble in aqueous media, and exhibits no cytotoxicity at very high concentrations (1000 μM) [229]. Substitution of the arginine residue for a glutamate residue results in the negative version of SAP, SAP(E) ((VELPPP)3), the first anionic CPP, which retains its propensity to adopt a PII helical secondary structure and is not cytotoxic at high concentrations (1000 μM). Since SAP(E) is negatively charged at physiological pH, the initial electrostatic interaction with the cell membrane is not necessary for its internalization. It is hypothesized that the mechanism of entry involves an initial aggregation on the cell surface, leading to an internalization mechanism that is independent of clathrin and probably caveolin mediated [231,235]. The chemical modification of SAP(E), by adding the Ac-CGGW sequence to the N-terminus of the primary structure, allows attachment to biologically active substances. For example, this linker contains an activated thiol and aminooxy functionality capable of generating a stable oxime bond, required for drug modification at the terminal cysteine, with the C-13 carbonyl group of doxorubicin. Cellular uptake and cytotoxicity studies in MCF-7 and HT-29 cancer cells demonstrated that this CPP–drug conjugate can efficiently transport doxorubicin through the cell membrane. The conjugate can be efficiently cleaved by glutathione within a short period, delivering the toxic cargo into the nucleus [236]. This delivery system solves the serious problem of passage through membranes, as doxorubicin’s anticancer activity targets DNA intercalation and topoisomerase inhibition [237].

##### Bac-7

Bac-7 (RRI RPRPPRLPRPRPRP LPFPRPGPRPIPRP LPFPRPGPRPIPRP LPFPRPGPRPIPRP), an antimicrobial proline-rich peptide, with four 14-residue repeats from the bactenecin family, is divided into five distinct regions: a charged cap (RRI), a degenerated repeat (RPRPPRLPRPRPRP), and three copies of a 14-residue repeat ((LPFPRPGPRPIPRP)3) [238]. Shortened Bac-7 fragments, regardless of their charge or hydrophobic content, penetrate cells and lack membranolytic activity. The cell-permeant antimicrobial activity of Bac-7 is localized in the N-terminal residues 1-24 (Bac1-24). This sequence is amphipathic and displays segregated charge and hydrophobic areas in three distinctive regions [232]. This proline-rich peptide was able to deliver a non-covalently bound protein to cells, indicating that Bac-7 and its derivatives are highly efficient CPPs for the intracellular delivery of proteins or peptides. For example, in a study to develop a thermally responsive polypeptide inhibitor of c-Myc, the cellular uptake, intracellular distribution, and potency of the Pen, Tat, and Bac1-24 cell-penetrating peptides fused to ELP (Elastin-Like Polypeptide)-H1 (a peptide which blocks c-Myc/Max dimerization) were evaluated. The conjugate Bac1-24-ELP-H1 localized to the nucleus of a subset of the cells and was the most potent MCF-7 cell proliferation inhibitor, compared to the CPPs Penetratin and Tat, resulting in a more potent c-Myc inhibitory polypeptide [239]. Massodi et al. developed a polypeptide carrier for a cell cycle inhibitor peptide, using a similar strategy. The coding sequence of ELP was modified by adding the CPP Bac-71-24 at the N-terminus and a 23-amino acid peptide derived from p21 at the C-terminus (Bac1-24-ELP1-p21). Bac1-24-ELP1-p21 displayed both cytoplasmic and nuclear distribution in SKOV-3 cells, inducing caspase activation, PARP cleavage, and cell cycle arrest in the S-phase and G2/M-phase [240]. These studies suggest that such macromolecular biopolymers with antiproliferative activity have great potential in cancer therapy for the targeted treatment of solid tumors.

##### Poly-L-Proline Type II Helix (PPII) Based

A specific class of CPPs is based on polyproline secondary structures, the poly-L-proline type II helix (PPII). PPII has a left-handed helical structure with distinct trans isomers of peptide bonds with dihedral angles of [−75°, +150°]. The rise per residue of the PPII helix is 3.1 Å with three residues per turn, resulting in a helical structure rising 9.3 Å per turn compared to the 6.0 Å pitch of a 310 helix. The primary reason for such an open and elongated geometry of PPII is the absence of H-donor atoms due to the cyclic side chain of proline residues. The PPII conformation is highly acceptable of H-donor atoms from its environment or third-party moieties, enhancing its solvation energy [241,242,243]. This architecture allows for the precise orientation of hydrophilic and hydrophobic moieties along different faces of the helix, making the structure soluble in aqueous media. To ensure that the designed CPPs adopt a PPII helical structure, the key is to maintain a proline content of at least 50%. These structural characteristics have been used in the design of CPPs with a rigid secondary structure, such as proline-based dendrimers [233], proline-derived γ-peptides like cis-γ-amino-L-proline [244,245], and cationic amphiphilic polyproline helices (CAPHs) [246,247,248,249]. Proline-rich peptides are highly water soluble, an invaluable property for life science applications.

### 2.3. Hydrophobic CPPs

The design of a new class of peptides, the hydrophobic ones, aims not only to mimic but also to improve the translocation properties of known peptides. To achieve this goal, the design considers various parameters such as electrostatics, secondary structures, and hydrophobic properties. Hydrophobic peptides are characterized by having only non-polar residues, a low net charge (less than 20% of the sequence), and a hydrophobic motif or chemical group that is critical for uptake independent of the rest of the sequence. These peptides are subdivided into two types: linear hydrophobic peptides based on natural amino acids and chemically modified peptides, including stapled peptides, prenylated peptides, and pepducins.

#### 2.3.1. Linear Hydrophobic Peptides Based on Natural Amino Acids

##### Pentapeptides (CPP5)

Linear hydrophobic peptides are formed by combining sequences of up to 70% to 90% aliphatic residues with aromatic, anionic, or cationic residues (10% to 30%). These peptides typically consist of pentapeptides (CPP5) and serve as the minimal cell-penetrating sequence within longer CPPs. An example of such CPP5s is derived from the Bax-binding domain of Ku70, known as Bax-inhibiting peptides (BIPs). These antiapoptotic peptides were designed based on the Bax-binding domain of Ku70 found in various species, including humans, rats, and mice. The CPP5s derived from this domain include VPMLK (V5 antiapoptotic pentapeptide), PMLKE, VPTLK, VPALR, and VPALK. Among these, VPTLK and KLPVM demonstrated protein-transduction activity [250]. Evaluation of the protein transduction activity of these two pentapeptides in cell culture revealed that, when VPTLK and KLPVM were added to the N-terminus of the Cre protein, the complexes were able to activate the expression of the Cre-inducible GFP gene, suggesting that the Cre complexes were able to reach the chromosomal DNA in the nucleus [251].

The cytoprotective activities of various other BIPs have been previously reported in both cell culture and animal models. For instance, the V5 antiapoptotic pentapeptide (VPMLK) was shown to prolong the survival of mice with acute liver failure when they were transplanted with monkey hepatocytes previously cultured with V5. V5 aids in increasing the survival of isolated hepatocytes and protects the cells by enhancing the expression of CAS. Thus, the action of V5 on monkey hepatocytes can be considered to be a stimulation of the kinase cascade and its complex, resulting in the stabilization of hepatocytes against stress signals [252]. Hepatocyte transplantation (HTX) is a potential treatment for liver failure and inborn errors of liver metabolism. The results of recent studies suggest that V5 could be used to administer other molecules that facilitate cell growth and proliferation or decrease the cellular immune response in target cells in other diseases.

For example, pancreatic islet transplantation holds great promise as a treatment for type 1 diabetes but often requires transplantation of islets from two to four donors. Following transplantation, islets undergo apoptosis and necrosis from transient hypoxia, lack of nutrient support, and hyperglycemia-induced toxicity. V5 inhibits a wide range of caspases, improving pancreatic islet recovery. This molecule binds Bax and prevents mitochondrial cytochrome c translocation, resulting in the global inhibition of caspases through the activation of NF-κB–dependent and BH1–4 genes [253]. The use of V5 in treating isolated islets increases their viability, improves their function, and prevents apoptosis. When islets are transplanted together with fibroblast growth factor-2 (FGF-2) and V5, it becomes possible to use a smaller mass of islets from a single donor pancreas for transplantation [254]. The approach described above effectively restores normal blood sugar levels and preserves insulin content and islet function after transplantation. Based on their findings, the authors suggest that exploring a timed release of V5, possibly achieved through gelatinization, could be a viable strategy to enhance long-term prevention of apoptosis and further improve outcomes in human islet transplantation.

##### Pep-7, SG3 and FGF

Several studies using phage and plasmid display techniques have identified a significant number of unusual, non-amphipathic, and minimally charged peptides that could potentially be classified as hydrophobic CPPs. Some examples include Pep-7 (SDLWEMMMVSLACQY) [255], SG3 (RLSGMNEVLSFRWL) [256], and FGF (PIEVCMYREP) [257]. These peptides have approximately 60%, 57%, and 60% apolar residues, respectively, and exhibit a net charge ranging from +2 (Pep-7) to +1 (SG3). Further investigations are necessary to elucidate the functions of these peptides and determine whether their hydrophobicity or other factors influence their absorption properties.

#### 2.3.2. Stapled Peptides

Hydrocarbon stapling, pioneered by Schafmeister et al. in 2000, is a chemical strategy developed to create a novel class of hydrophobic CPPs, known as stapled peptides. Initially, this method used α,α-disubstituted non-natural amino acids with olefin-bearing tethers to generate an all-hydrocarbon “staple” through ruthenium-catalyzed ring-closing metathesis (RCM). This hydrocarbon stapling approach stabilizes the α-helical structure of peptides, enhancing their membrane permeability and proteolytic stability, thereby improving their potential as therapeutic agents [258].

In all cases, the peptide stapling technique involves cross-linking the side chain of an amino acid residue to either the peptide terminus or another side chain within the native peptide. This ensures that the selected or substituted amino acids are positioned on the same helical face, allowing covalent cross-linking [259,260,261,262]. In α-helices, residues align along the same helical face every 3.6 residues. Specific positions for stapling include i/i + 3, i/i + 4 (one-turn staple), i/i + 7 (two-turn staple), and i/i + 11 (three-turn staple). Covalent connections at these positions stabilize the helical conformation, enhancing structural integrity and proteolytic stability. Various chemical reactions have been employed to achieve these cross-links, broadening the applicability of peptide stapling in drug development [259,263,264,265]. Peptide stapling produces peptides with superior binding affinity, selectivity, and enhanced metabolic stability compared to their unmodified counterparts. This technique not only increases the bioavailability of peptide drugs but also extends their half-life, providing a robust solution for accelerating the development and clinical application of peptide-based therapeutics [266,267,268,269].

Stapled peptides offer increased proteolytic stability and enhanced cell permeability, addressing key limitations of conventional peptides and making them highly effective as therapeutic agents. By maintaining a stable α-helical structure in biological environments, they are particularly useful for modulating intracellular pathways. These advantages have demonstrated significant potential in targeting protein–protein interactions, which are often challenging for traditional small molecule drugs. Stapling increases peptide helicity by rigidifying the peptide structure and fortifying the natural α-helical structure that would otherwise unfold outside the context of the host protein. This is important because the α-helix, a major structural motif of proteins, often mediates intracellular protein–protein interactions that govern many biological pathways and enhances the cell penetration capacity of various CPPs.

##### SAHBs

This chemical strategy has been used to generate BH3 peptides with improved pharmacological properties. The stapled peptides, called stabilized α-helix of BCL-2 domains (SAHBs), proved to be helical, protease-resistant, and cell-permeable molecules that bind with increased affinity to multidomain BCL-2 member pockets. A SAHB of the BH3 domain from the BID protein, SAHB_*A*_ (EDIIRNIARHLA(S5)VGD(S5)NLDRSIW), specifically activated the apoptotic pathway to kill leukemia cells. SAHB_*A*_ effectively inhibited the growth of human leukemia xenografts in vivo. Histological examination of SAHB_*A*_-treated mice showed no toxicity of the compound to normal tissue [270]. The hydrocarbon stapling technique applied to BH3 death domain peptides has been instrumental in addressing the shortcomings of CPPs, such as the loss of their bioactive structure in solution, rapid proteolytic degradation in vivo, and limited cell permeability [271].

Researchers have utilized the natural activity of the Bcl-2-interacting mediator of cell death (BIM), which interacts with BCL-2 and possesses one of the most potent BH3 death domains in the BCL-2 protein family, to attempt to restore BH3-dependent cell death in resistant hematological cancers. A notable example of this approach comes from the work of LaBelle and colleagues. Their studies demonstrate that BIM-SAHB_*A*_, a stapled BIM BH3 helix that contains an i, i + 4 all-hydrocarbon cross-link spanning positions 154 and 158 (designated “A”), effectively reactivates cell death in vitro and in vivo in a BH3 sequence-dependent manner. Furthermore, BIM-SAHB_*A*_ blocks the antiapoptotic sequestration of BAX/BAK BH3 helices, leading to the release of mitochondrial cytochrome c in a BAX/BAK-dependent manner. The treatment with BIM-SAHB_*A*_ also activates caspase-3/7 and induces cell death in resistant hematologic cancer cells [272]. An interesting example involves the complex of MCL-1 and the peptide SAHB_*D*_ (EDIIRNIAR(R5)LAQVGD(S8)NLDRSIW). This complex effectively targets native MCL-1, disrupting its capacity to suppress the death pathway through protein interactions. Consequently, it sensitizes caspase-dependent cancer cell apoptosis when death receptor stimulation occurs. The evidence obtained has enabled the authors to develop a model for the creation of novel therapies aimed at reactivating apoptosis in diseases driven by pathological cell survival mediated by MCL-1 and chemoresistance [273].

##### NYAD-1

Another case of interest is the modified peptide NYAD-1, derived from a 12-mer α-helical peptide (CAI). In cell culture, the original peptide (CAI) failed to inhibit HIV-1 due to its inability to penetrate cells. However, NYAD-1, with enhanced α-helicity through molecular stapling chemical modification, is capable of cell penetration without the need for a carrier protein. It disrupts the formation of both immature- and mature-like HIV-1 particles and effectively inhibits HIV-1 infection in cell cultures. NYAD-1 demonstrates high-affinity binding to the C-terminal domain of the capsid and holds potential as a new class of drugs for AIDS treatment [274].

##### ALRN-6924

ALRN-6924 (Sulanemadlin: AcLTF(R8)EYWAQL(S5)AAAAA(dA)-NH2, with a staple between R8 and S5) is a stapled peptide designed to mimic the N-terminal domain of the p53 tumor suppressor protein. This peptide exhibits high-affinity binding to MDM2 and MDMX (MDM4), the natural inhibitors of p53, thus activating p53 signaling in cells with a wild-type TP53 genotype (TP53-WT). Ingelshed et al. have demonstrated that MDM2/MDMX inhibition by sulanemadlin reduces cell growth in a p53-dependent manner. They also showed that p53 activation by sulanemadlin increases the expression of immunogenicity markers, enhances lymphocyte infiltration when combined with anti-PD-1 immunotherapy, and synergistically improves overall survival [275]. Through iterative structure–activity relationship (SAR) optimization, ALRN-6924 has been engineered to possess favorable characteristics in terms of cell permeability, solubility, pharmacokinetics, and safety. The intracellular proteolysis of ALRN-6924 results in a long-acting metabolite that maintains a strong binding affinity to MDM2 and MDMX, with slow dissociation kinetics. At high doses (10 mg/Kg), ALRN-6924 exhibits mechanism-based anticancer activity in TP53-WT tumor models. Conversely, at lower doses, it transiently arrests the cell cycle in healthy tissues, thereby providing chemoprotection without affecting TP53-mutant cancer cells [276]. ALRN-6924 activates the transcription factor p53, resulting in a unique pharmacodynamic response. Saleh, M. N., et al., have shown that after ALRN-6924 administration, serum levels of MIC-1, a protein regulated by p53, increased rapidly and remained elevated for more than 48 h at the RP2D, indicating sustained p53 activation. This finding suggests that effective therapies can achieve prolonged effects despite the drug’s plasma half-life of 5.4 h [277]. Recent reports indicate that ALRN-6924 enhances the antitumor efficacy of chemotherapy in TP53 wild-type hormone receptor-positive breast cancer models. Pairawan et al. demonstrated that ALRN-6924 is active in WT-TP53 cancer cell lines but not in mutant TP53. In ER+ breast cancer cell lines, it showed synergistic effects in vitro and improved in vivo antitumor activity with paclitaxel and eribulin. Apoptotic assays revealed a significantly higher in vivo apoptotic rate with the combination of ALRN-6924 and paclitaxel compared to either agent alone, warranting further evaluation in hormone receptor-positive breast cancer patients [278]. In preclinical studies, ALRN-6924 significantly improved survival outcomes in an animal model of human acute myeloid leukemia (AML). Mice transplanted with human leukemia cells exhibited a threefold increase in median survival, from 50 days to approximately 150 days, highlighting the therapeutic potential of ALRN-6924 (NCT02909972) [279]. These and other findings have led to the conduct of multiple clinical trials to investigate the potential of ALRN-6924, both as a monotherapy and in combination, for the treatment of various diseases (NCT02264613, NCT04022876, NCT03654716, NCT05622058, NCT02264613) (see Table 1).

Some of these CPPs have been shown to enter cells via an endosomal mechanism, rather than a membrane rupture mechanism [280]. Studies on the application of molecular stapling to multiple cell targets have revealed several key findings. First, the stapled peptides are localized in the mitochondria and multivesicular bodies of intact cells. Second, the stapled peptides exhibit reduced binding affinity and poor cell permeability, which may lead to a loss of activity [281]. Molecular stapling, in some cases, has been found to increase cytotoxicity. Several studies indicate that certain constrained peptides can induce cell lysis by rupturing the cell membrane, which is undesirable and likely leads to non-specific toxicity [282,283]. The molecular stapling technique has been applied in various contexts. For a more comprehensive review, it is advisable to consult reports related to the generation of stapled peptides for high-affinity protein binding [284], intracellular targeting [270,273], binding site discovery [285,286,287], potential therapeutic applications in cancer [288,289,290], diabetes [291], vaccine development [292], and viral infection [293]. In the context of the COVID-19 pandemic, some research groups have explored the possibility that stapled peptides can inhibit the binding of SARS-CoV-2 to ACE2 receptors [294,295]. However, one of these peptides was reported to have no antiviral activity against SARS-CoV-2. Additionally, further studies are needed to improve the ability of stapled peptides to block viral entry [296,297].

In summary, hydrocarbon stapling is a potent technique in peptide drug development, providing a method to improve the pharmacokinetic properties and therapeutic potential of peptides. The ongoing evolution and optimization of this technology promise novel treatments for a broad spectrum of diseases.

#### 2.3.3. Prenylated Peptides

The prenylation of peptides involves adding a lipid chain, consisting of three isoprene units (farnesyl) or four isoprene units (geranylgeranyl), to a free thiol group found in specific Cys residues near the C-terminus of a protein. This addition of either a farnesyl (C15) or geranylgeranyl (C20) isoprenoid moiety has been shown to confer inherent cell-penetrating capacity to peptides. These peptides can efficiently cross the cell membrane through an ATP-independent, non-endocytic pathway, leading to their accumulation in the cytosol [298,299]. Prenylated peptides have potential applications in cell-penetrating therapies, especially when the cargo needs to be readily released in the cytosol. It is worth noting that the specific sequence of the peptide does not significantly affect uptake, as long as the geranylgeranyl group is present [300,301]. Various studies have demonstrated that prenylated peptides are internalized via a non-endocytic pathway, irrespective of their sequence. Additionally, both the length and identity of the sequence can influence peptide uptake, with even short prenylated sequences containing just two amino acids exhibiting significant cell-penetrating properties [299].

Natural prenylated indoles have been proposed as potential anticancer agents. The design of a novel pentapeptide sequence, including a prenyl residue in one of the tryptophan residues (N-tert-prenylated tryptophan), was based on the Substance P Antagonist G (Arg–d-Trp–NMePhe–d-Trp–Leu–Met-NH2), a known anticancer agent for small cell lung cancer (SCLC). The resulting prenylated peptide showed favorable cytotoxicity against H69 and DMS79 SCLC cell lines when compared with the unmodified pentapeptide or the original SPG sequence. In vivo studies demonstrated that the pentapeptide exhibited antitumor activity at relatively low doses (1.5 mg/kg) against the growth of the DMS79 xenograft and remained stable in plasma for at least 3 h [27].

#### 2.3.4. Pepducins

Pepducins are cell-penetrating, membrane-tethered lipopeptides designed to target the intracellular region of various transmembrane proteins, such as GPCRs and MMPs. Depending on the peptide sequence, they can act as agonists, antagonists, or modulators of the protein’s activity. Pepducins consist of a lipid moiety, such as myristate, palmitate, or lithocholic acid, which enables them to easily penetrate cell membranes. This lipid is attached to a peptide corresponding to an amino acid segment from one of the cytoplasmic loops (intracellular i1–i3 domains) or the C-terminal tail (intracellular i4 domain) of the target GPCR. In the presence of their cognate receptor, pepducins activate receptor G-protein signaling and exhibit high selectivity for the receptor type. Pepducins are commonly modified at the N-terminus, and both the lipid moiety and amino acid sequence can be optimized to achieve the desired pharmacological and pharmaceutical properties. While pepducins are believed to function as allosteric modulators of GPCR signaling by binding to the intracellular surface of target receptors, the precise pharmacological basis of their activity remains unknown [28,302,303,304].

In the orthosteric activation mechanism of a GPCR, an agonist binds to the orthosteric binding pocket located within the receptor’s transmembrane bundle near the extracellular surface. This binding triggers a conformational change in the receptor, leading to the externalization of part of TM6 and rotation of TM7. As a result, an ionic lock between TM3 and TM6 is broken. The E(D)RY sequence in helix 3 facilitates proton uptake, stabilizing the active receptor conformation. In this state, the receptor’s intracellular portion exposes a predominantly hydrophobic cavity, allowing the C-terminal helix of the G-α protein to engage and exchange GDP for GTP [305,306,307]. Allosteric inhibition, which involves the modulation of signaling through a site other than the orthosteric ligand binding site, offers new drug-targeting opportunities. Pepducins can access the intracellular face of GPCRs chemically and physically, enabling the identification of both orthosteric and allosteric modulators for these significant drug discovery targets [308].

Due to the above, cell-penetrating lipidated peptides represent a class of compounds that can be used as novel tools to probe complex mechanisms of diverse GPCR activity [309] and other membrane proteins, including muscarinic acetylcholine receptor, chemokine receptors (CXCR1, CXCR2, and CXCR4) [310,311], protease-activated receptors (PAR1, PAR2, and PAR4) [312], the melanocortin-4 receptor, the Smoothened receptor, formyl peptide receptor-2 (FPR2), the relaxin receptor (LGR7), sphingosine 1-phosphate receptor-3 (S1P3), G-proteins (Gαq/11/o/13), and the GPIIb integrin. Pepducins are being investigated as a potential treatment strategy for various human diseases, including cardiovascular diseases [302,313,314,315,316,317], cancer [312,318,319,320,321], inflammation, sepsis [322,323,324], asthma [304], and bone marrow transplant [325].

##### P1pal-7

P1pal-7, also known as PZ-128, is a 7-mer palmitoylated pepducin (palmitate-KKSRALF, PAR1 pepducin) that functions as a reversible inhibitor of the protease-activated receptor 1 (PAR1) on platelets and other vascular cells. This inhibition is achieved by targeting the intracellular surface of the receptor, thereby preventing signal transduction associated with platelet activation and other cellular responses. Structurally, PZ-128 mimics the predicted off-state conformation of the juxtamembrane region of the third intracellular loop of PAR1, effectively blocking its activation. It targets the cytoplasmic surface of PAR1 and interrupts signaling to internally located G proteins [302,303,323,326]. The efficacy of PZ-128 as an antiplatelet agent has been demonstrated in vivo, where it significantly reduces ischemic events by inhibiting platelet aggregation and thrombosis [317]. In clinical studies, PZ-128 has progressed successfully through phase 2 trials in cardiac patients, demonstrating safety and efficacy in reducing myonecrosis and arterial thrombosis (NCT01806077, NCT02561000) (see Table 1) [327]. The advancement of PZ-128 underscores its potential as a therapeutic agent in cardiovascular diseases, providing a novel approach to antiplatelet therapy by specifically targeting the intracellular mechanisms of PAR1. Beyond cardiovascular applications, PZ-128 shows promise in cancer and systemic inflammation. In cancer studies, nude mice were inoculated in their mammary fat pads with the invasive breast cancer cell line MCF7-PAR1/N55 and treated with either a vehicle or the PAR1 pepducin, P1pal-7. By the end of the treatment period, P1pal-7 significantly inhibited the growth of MCF7-PAR1/N55 xenografts by 62% (*p* < 0.01). Tumors excised from the mammary pads revealed extensive replacement of normal mammary tissue upon hematoxylin and eosin staining. Moreover, P1pal-7 treatment resulted in a significant reduction in blood vessel density at the center of the tumors by 75% (*p* < 0.002) [312].

Pepducins represent a promising class of cell-penetrating peptides with significant therapeutic potential. Their unique mechanism of action, targeting the intracellular surface of GPCRs, offers a novel approach to inhibiting receptor-mediated signaling. Extensive preclinical testing has shown that pepducins possess favorable pharmacodynamic and pharmacokinetic properties, along with suitable biodistribution and low-toxicity profiles. Moreover, their simplicity in synthesis and purification in large quantities makes them viable candidates for drug development [328]. These findings underscore the potential of pepducins as versatile therapeutic agents in the treatment of various diseases, including cancer, cardiovascular conditions, and systemic inflammation [329,330,331].

### 2.4. Cyclic CPPs (cyCPPs)

Cyclic cell-penetrating peptides (cyCPPs) have garnered substantial interest within the scientific community for their exceptional ability to traverse cellular membranes, representing a pivotal advancement in the field of biomedical research [332,333,334,335]. The synthesis of cCPPs leverages advanced peptide chemistry strategies in conjunction with solid-phase synthesis techniques. This process systematically forms peptide bonds between amino acids, progressively lengthening the peptide chain. The defining feature of cCPPs is their distinctive cyclic structure, which is achieved by the strategic insertion of a linker or bridge between the N- and C-termini, resulting in a closed-loop formation [336,337]. The establishment of a covalent bond between the peptide’s amino and carboxyl ends enhances its stability, provides significant resistance to enzymatic degradation, and markedly improves its cellular uptake capabilities [338,339]. To further enhance these properties, an array of chemical modifications are employed. Methods including cyclization through disulfide bonds or other cross-linking approaches are utilized to improve the stability and effectiveness of cCPPs [340]. Specifically, peptides can be made cyclic by creating disulfide or lactam bridges or through the modification and cyclization of the peptide main chain for increased performance [341]. Recent advancements have led to the development of methodologies that facilitate the rapid construction of extensive cyclic peptide libraries [342,343,344,345], broadening the spectrum of potential inhibitors for targets that were previously considered challenging to address with drug-based interventions [343].

Numerous studies have demonstrated that several cyclic CPPs directly interact with phospholipids on the plasma membrane, facilitating their cellular entry through endocytosis while exhibiting minimal cytotoxic effects [332]. Endosomal escape studies suggest an alternative cargo release mechanism, where CPPs directly bind to the luminal side of the endosomal membrane, inducing curvature and forming CPP-enriched small vesicles. This process involves significant membrane distortion, marked by sharp negative Gaussian curvatures, representing the highest energy transition state. CPPs selectively bind to budding necks, reducing the energy barrier and accelerating budding. Endosomal acidification enhances this process by increasing the affinity of arginine-rich CPPs for the membrane. Upon detachment from the endosomal membrane, small vesicles likely become less stable due to the dissipation of pH gradients across their membrane, resulting in luminal content release into the cytosol [332]. pH gradients across artificial membranes can persist for minutes to hours, indicating efficient proton movement across lipid bilayers [346]. The late endosome membrane, rich in negatively charged lipids like bis(monooleoylglycero) phosphate (BMP) and phosphatidylinositol (PI), further facilitates cationic CPP binding, promoting vesicle budding and concentrating CPPs within budding vesicles.

Compared to linear CPPs, cyCPPs exhibit notable advantages, including enhanced structural stability, superior cellular penetration capabilities, and reduced susceptibility to intracellular proteolysis [332,336,347]. These attributes position cyCPPs as promising candidates for the efficient delivery of drugs, therapeutic molecules, therapeutic approaches known as Peptide Receptor Radionuclide Therapy (PRRT), and imaging probes across a myriad of biomedical applications. Firstly, cyCPPs excel in drug delivery, efficiently ferrying therapeutic agents ranging from small-molecule drugs to peptides and nucleic acids such as siRNA, DNA, and RNA. This capability is invaluable for addressing diseases requiring precise intracellular targeting [348,349,350,351,352,353,354,355,356,357]. Moreover, cCPPs play a pivotal role in imaging applications, where they can be conjugated with imaging agents like fluorophores and radioisotopes. This conjugation enhances cellular and tissue imaging, significantly advancing diagnostic capabilities and research endeavors [118,358,359,360,361,362]. Additionally, in gene therapy, cyCPPs are instrumental in facilitating the delivery of gene-editing tools and therapeutic genes, enabling precise manipulation of cellular functions for therapeutic purposes [363]. Lastly, cyCPPs serve as indispensable research tools in both cell biology and molecular biology, facilitating the study of intricate cellular processes and protein functions, driving advancements in scientific understanding and therapeutic innovation [364,365].

In drug design, cyCPPs have emerged as potent inhibitors of protein–protein interactions (PPIs). These interactions, characterized by intricate interfaces spanning substantial surface areas—typically between 1000 and 5000 Å^2^—pose a challenge to conventional small molecule approaches. The distinctive attributes of cyCPPs, including their resistance to chemical or enzymatic degradation and their heightened receptor selectivity [338], render them particularly promising. The utilization of these innovative CPPs has significantly broadened the spectrum of potential inhibitors for targets previously deemed “undruggable”. In this context, cyclic peptides are gaining traction as a promising foundation for drug development, offering novel avenues to enhance drug delivery and efficacy. These peptides, along with cyclic depsipeptides and bicyclic peptides found in nature, exhibit a wide array of chemical structures. Known for their diverse biological effects—ranging from fighting cancer, bacteria, viruses, and fungi to reducing inflammation and clotting—cyclic peptides and their derivatives underscore their importance in the search for new pharmacological agents, driving ongoing research into their synthesis, characterization, and clinical applications [366].

#### 2.4.1. BT1718

BT1718 exemplifies a Bicycle Toxin Conjugate (BTC), a novel class of therapeutics that leverages bicyclic peptide technology for targeted drug delivery. This conjugate comprises a bicyclic peptide that binds with high affinity and selectivity to the hemopexin domain of membrane type 1 matrix metalloproteinase (MT1-MMP) [29]. MT1-MMP is critically involved in cell invasion and metastasis and is often overexpressed in solid tumors associated with poor patient prognosis [367,368,369,370]. The peptide component of BT1718 is connected via a molecular spacer and a cleavable disulfide linker to DM1 (N2′-deacetyl-N2′-[3-mercapto-1-oxopropyl]-maytansine), a potent cytotoxic tubulin inhibitor.

BT1718 targets cells expressing MT1-MMP, exploiting its overexpression on tumor cells to deliver the cytotoxic payload specifically to the tumor site. The conjugate’s design ensures that once the linker is cleaved in the tumor microenvironment, the active DM1 is released, binding to microtubules and inhibiting tumor cell division, leading to cell death and tumor size reduction. Unlike traditional MMP inhibitors, BT1718 does not inhibit MT1-MMP activity but uses it as a delivery mechanism for its cytotoxic cargo [29].

BT1718 has demonstrated promising efficacy against treatment-resistant cancer samples. Additionally, it has shown reduced toxicity compared to other potent cancer treatments, highlighting its potential as a safer and more effective therapeutic option for aggressive cancers. By specifically targeting MT1-MMP, BT1718 effectively induces tumor cell death and inhibits tumor growth while minimizing toxicity. The promising preclinical results and favorable safety profile of BT1718 position it as a highly innovative and effective therapeutic candidate for treating aggressive and treatment-resistant cancers (NCT03486730) (see Table 1).

#### 2.4.2. ^177^Lu-DOTA^0^-Tyr^3^-Octreotate

^177^Lu-DOTA^0^-Tyr^3^-octreotate is a radioconjugate comprising the somatostatin analog Tyr3-octreotate (TATE) conjugated to the chelating agent DOTA and radiolabeled with the β-emitting isotope lutetium-177 (^177^Lu). This radioconjugate possesses both imaging and antineoplastic capabilities, demonstrating high-affinity binding to type 2 somatostatin receptors (SSTR2s) expressed on neuroendocrine tumor (NET) cells. Upon receptor binding and subsequent internalization, it delivers a cytotoxic dose of β radiation directly to SSTR2-positive cells, thereby selectively targeting and eradicating these tumor cells. ^177^Lu-DOTA^0^-Tyr^3^-octreotate represents a significant advancement in PRRT. It demonstrates superior tumor uptake and extended residence times while maintaining reduced whole-body retention, thereby mitigating the risk of bone marrow toxicity and preserving renal function post-PRRT [371,372]. Furthermore, the use of renal protective agents significantly reduces adverse effects, which are generally mild, with therapy responses extending beyond two years. Its radioligand properties facilitate the real-time monitoring of treatment efficacy and enable precise dose adjustments [373,374,375,376]. Numerous clinical trials have been conducted to assess its efficacy, both as a monotherapy and in combination with other agents, for the treatment of various conditions (see Table 1), including pulmonary neuroendocrine tumors (NCT03325816) [377], bronchial and gastroenteropancreatic neuroendocrine tumors [378], and progressive neuroendocrine tumors [379], among others. Approved by the FDA as Lutathera^®^ in January 2018 and by the EMA in September 2017, it is the first radiopharmaceutical agent indicated for the treatment of unresectable or metastatic, progressive, well-differentiated (G1 and G2) SSTR-positive gastroenteropancreatic neuroendocrine tumors (GEP-NETs) in adults [380,381].

## 3. The CPPs Classification by Their Origin

### 3.1. Protein-Derived CPPs

Natural proteins contain valuable structural motifs that serve as CPPs. These CPPs have the ability to enter cells independently, but they may not confer inherent cell-penetrating capabilities to the entire protein. Conversely, certain natural proteins may contain CPP sequences that do not have cell-penetrating abilities when isolated. The role of the CPP sequence within the full-length protein is often unknown, which adds complexity to understanding cell-penetrating mechanisms.

In this section, CPPs are classified based on the originating group of proteins or peptides. In certain instances, the cell-penetrating ability of CPPs is directly linked to the function of the parent protein or peptide. However, this correlation is not universally observable. Despite numerous reports documenting biologically active CPPs, the exact pharmacological mechanisms underlying their activity often remain unclear, even after thorough characterization of the source proteins. This ambiguity frequently arises from insufficient biochemical and mechanistic data.

#### 3.1.1. Homeoprotein-Derived Cell-Penetrating Peptides

Homeoproteins are a family of transcription factors that contain a conserved DNA-binding motif called a homeodomain, which plays a crucial role in regulating the subcellular localization of these proteins. The homeodomain is a 60-amino acid DNA-binding domain that exhibits high conservation across homeoproteins and species, adopting a three-helical fold with a characteristic helix-turn-helix topology [382]. The presence of the homeodomain defines the homeoprotein family, with its molecular signature being the third α helix that exactly corresponds to the Penetratin motif. This third helix is responsible for recognizing the DNA binding site, serving as the structural fragment that binds to the main groove of DNA [383]. More than 300 members of homeoproteins have been identified in humans, and many of them demonstrate efficient translocation through biological membranes. Some extensively studied homeoproteins include Antennapedia [384,385], HoxA-5 [386], Engrailed [387], pIsl peptide [388], and PDX-1 [389], among others.

To analyze the effects of sequence divergence in the homeodomain, several authors compared the cellular uptake efficiencies and interaction properties of four peptides corresponding to the third helix sequence of Antennapedia, Engrailed-2, HoxA-13, and Knotted-1 in a membrane-mimicking environment. Although all these peptides were found to translocate into cells, significant differences in their uptake efficiencies were observed. As expected, these peptides adopt helical conformations and are positioned parallel to the surface of the micelle. However, subtle differences in immersion depth were noticed among them. Interestingly, the peptide with the highest uptake efficiency was the least deeply inserted within the micelle, suggesting that electrostatic surface interactions may play a major role in membrane translocation [84]. These peptides, among others, have been widely used to internalize charges into the cytoplasm and nucleus of various cell types in vivo and in vitro. For example, the pIsl peptide, which corresponds to the third helix of the homeodomain from the rat insulin-1 gene enhancer protein, efficiently internalizes into human Bowes melanoma cells. The researchers demonstrated the ability of pIsl to carry large molecules into the cells by coupling biotinylated pIsl peptide to fluorescently labeled avidin, a biotin-binding protein with a molecular weight of approximately 63 kDa. The observations showed that pIsl, with a sequence analogous to that of penetratin, translocates into the cells in a nonendocytotic manner. Based on structural studies of pIsl, it has been suggested that utilizing the native Cys residue in the pIsl sequence may enable various coupling reactions. This characteristic makes pIsl a valuable candidate for the cellular delivery of diverse hydrophilic cargo molecules [388].

Homeodomain-derived CPPs have been used to block the function of specific transcription factors involved in the progression of different types of cancer. For example, the neural-specific transcription factor, Engrailed 1 (EN1), is exclusively overexpressed in extremely aggressive cancers. Experimental results have shown that the knockdown of EN1 triggered potent and selective cell death. In contrast, the ectopic overexpression of EN1 in normal cells activated cell survival pathways and conferred resistance to chemotherapeutic agents. This knowledge has been used to propose the inhibition of EN1 function as a potential strategy for containing cancer cell proliferation. To block EN1 function, synthetic interfering peptides (iPeps) have been designed, comprising the EN1-specific sequences that mediate essential protein–protein interactions required for EN1 function, and an N-terminal cell-penetrating peptide/nuclear localization sequence. These peptide conjugates (EN1-iPeps) elicited a strong apoptotic response in EN1-overexpressing tumor cells, with no toxicity to EN1-non-expressing or normal cells, effectively inhibiting its role as an activator of intrinsic inflammatory pathways associated with survival in basal breast cancer [390]. The engineering of homeoprotein-derived CPPs as a novel and selective therapeutic strategy is seen as a promising alternative to combat lethal forms of various types of cancer.

#### 3.1.2. Heparin Binding Proteins

Proteins that have an affinity for heparin or heparan sulfate (HS) serve as excellent sources of CPPs due to their ability to interact with cell surface receptors and effectively cross cell membranes. These proteins possess specific domains or motifs that enable them to bind to the highly abundant heparin or HS molecules present on the cell surface [391]. By leveraging these interactions, CPPs derived from such proteins can efficiently transport various cargoes, including drugs or therapeutic molecules, into the cell interior. This unique characteristic makes heparin/heparan-binding proteins highly valuable for the development of innovative strategies in biomedical and pharmaceutical applications, particularly for intracellular delivery.

The initial step for numerous CPPs in cell uptake involves binding to GAGs. Multiple studies have provided evidence of the interaction between negatively charged GAGs on the surface of biological cells and CPPs, resulting in the formation of clusters upon binding. This clustering phenomenon amplifies the endocytic uptake of CPPs. Proteins with GAG-interacting domains have been shown to serve as potential sources of CPPs. Based on this principle, a group of cationic CPPs, called Vectocell^®^ or Diatos Peptide Vectors (DPVs), was identified from human heparin-binding proteins and/or anti-DNA antibodies. These proteins include superoxide dismutase (DPV3 and DPV3/10), platelet-derived growth factor (DPV6), epidermal-like growth factors (DPV7-DPV7b), intestinal mucin (DPV10/6), apolipoprotein B (DPV1047), and cationic antimicrobial protein of 37 kDa (CAP 37) [392]. DPVs possess the ability to effectively facilitate the internalization of molecules ranging from a few Daltons to as large as 200 kDa. The internalization of DPVs (excluding DPV1047) relies on their interaction with GAGs present on the cell surface. The uptake mechanism of DPVs-GAG complexes involves active caveolar endocytosis, a pathway associated with signal transduction and the intracellular transport of lipid raft-associated molecules. Importantly, the utilization of different peptides enables the delivery of molecules to either the cell nucleus or cytoplasm.

A study was conducted to enhance the therapeutic index of doxorubicin by chemically conjugating it with short peptide sequences (15 to 23 amino acids) from the Vectocell family. The efficacy of these conjugates using different linkers was assessed. The results of the investigation showed that the therapeutic index of doxorubicin in vivo could be improved by linking it to DPV1047 using an ester linker at the C14 position of doxorubicin, in both colon and breast tumor models. The conjugated form of doxorubicin was more effective in treating tumors while reducing its toxicity to normal cells. Furthermore, the doxorubicin-peptide conjugate demonstrated significant in vivo antitumor activity in a model of doxorubicin-resistant cancer, indicating its potential to overcome the multidrug resistance (MDR) phenotype. This is an important finding, as MDR is a common challenge in cancer treatment where cancer cells become resistant to multiple drugs [393].

Subsequent investigations revealed that structurally diverse CPPs share the binding and clustering properties of GAGs. These investigations utilized techniques such as isothermal titration calorimetry and dynamic light scattering to measure GAG binding and clustering for different monovalent and multivalent CPPs. The results revealed microscopic dissociation constants in the range of 0.34 to 1.34 μM, which are biologically significant. The interactions between CPPs and GAGs led to the formation of aggregates with a hydrodynamic radius spanning from 78 nm to 586 nm, indicating multiple GAG chains interconnected by CPPs [20]. Remarkably, these binding and clustering characteristics were observed consistently across all examined CPPs. This valuable insight could guide the design of CPPs tailored for specific cellular uptake and internalization processes. Furthermore, the investigations have shed light on the potential sources of CPPs, which may be derived from proteins that naturally undergo internalization in cells. Moreover, the findings have enabled the identification of proteins exhibiting specificity toward particular cell types.

At the cellular level, it is well known that HS, a negatively charged molecule on the cell surface, plays a crucial role in the initial attachment of various basic CPPs through electrostatic interactions, leading to diverse cellular effects. The binding of HS molecules to specific sequences of basic amino acids, also known as Cardin–Weintraub or heparan sulfate/heparin-binding motifs, occurs in a structurally appropriate conformation. These motifs are further reinforced with other polar residues to enhance the stability of the complex [394,395]. In a study by Chen C. J. et al., a series of HS-binding CPPs derived from natural proteins were investigated, including CPPecp (NYRWRCKNQN). CPPecp was identified from a critical HS binding region in hRNase3, a unique member of the RNase family with known in vitro antitumor activity. Notably, CPPecp demonstrated multiple functions, such as strong binding activity to tumor cell surfaces with higher HS expression, significant inhibitory effects on cancer cell migration, and the ability to suppress angiogenesis both in vitro and in vivo [396].

#### 3.1.3. Viral Proteins

Numerous CPPs have been derived from motifs found in viral proteins that possess cell-penetrating abilities. One of the earliest CPPs discovered was the TAT peptide from the human immunodeficiency virus (HIV). The TAT peptide, rich in arginine and lysine residues and adopting an α-helical structure, has been extensively used in biomedical research and vaccine design [397,398].

Members of the Circoviridae family have also yielded CPPs like CVP1 and CVP1-N2, derived from the VP1 protein of the chicken anemia virus (CAV). These CPPs, containing multiple arginine residues and an α-helical structure, effectively transport plasmids and proteins into cells. The effectiveness of the CVP1 peptide as a macromolecule transporter was demonstrated through the successful delivery of β-galactosidase and nucleotides (plasmid and poly(I:C)) to cells. The data indicate that the cell-penetrating activity of CVP1 was significantly higher than that of TAT. Despite being used as a tool to deliver biomolecules for treating various diseases, including cancer, it has been documented that the TAT peptide sequence contains a motif recognized and cleaved by furin, compromising its stability and cell-penetrating ability when delivering exogenous payloads. Consequently, CVP1, lacking such a motif, could be considered a viable alternative to TAT for enhanced drug delivery or the delivery of active molecules, such as apoptin and functional siRNA, into cells for therapeutic applications in vivo [399,400,401]. On the other hand, CVP1-N2 exhibited even higher uptake efficiency compared to TAT. It effectively delivered the red fluorescent protein (RFP) and apoptin gene into cells, leading to apoptosis induction. CVP1-N2 may be suitable as a nucleic acid drug delivery tool in gene therapy applications [402,403].

In the same context, the potential of two new CPPs named pepR (Arg-rich CPP, LKRWGTIKKSKAINVLRGFRKEIGRMLNILNRRRR—residues 67-100 of DENV-2 C protein) and pepM (hydrophobic Pro-rich CPP, KLFMALVAFLRFLTIPPTAGILKRWGTI—residues 45–72 of DENV-2 C protein) has been examined. These CPPs were designed based on two domains of the dengue virus (DENV) capsid protein. The primary objective of the study was to investigate their ability to deliver nucleic acids into cells as non-covalently bound cargo. Translocation studies were conducted in various cell lineages, including HepG2, BHK, and HEK cells, as well as in astrocytes and peripheral blood mononuclear cells. The studies revealed distinct internalization routes for pepR and pepM: pepM demonstrated a direct translocation across lipid membranes, while pepR utilized an endocytic pathway. Both peptides preferentially bound to anionic lipid membranes, adopting an α-helical conformation. However, fluorescence quenching studies suggested that pepM is deeply inserted into the lipid bilayer, in contrast to pepR. Furthermore, both peptides successfully facilitated plasmid transfection, resulting in fully functional GFP protein expression. This indicates that while a portion of the cargo may be located in the membranes, a significant fraction is efficiently delivered [404].

Yamamoto et al. identified novel CPPs derived from the hemagglutinin cleavage site (pHACS) peptides of highly pathogenic influenza viruses. The study aimed to compare the CPP potential of peptides originating from the pHACS of four subtypes of influenza A virus (H1, H3, H5, and H7) and an influenza B virus (H1-pHACS, H3-pHACS, H5-pHACS, H7-pHACS, and B-pHACS). The data revealed that only the H5-pHACS and H7-pHACS peptides exhibited strong binding affinity to both mouse dendritic cells and human epithelial cells. These peptides were efficiently internalized into the cells, and the process required glycosaminoglycans, particularly HS and neuropilins, to bind effectively. The conducted studies further demonstrated that when H5-pHACS and H7-pHACS peptides were conjugated to antigens, they robustly induced the production of antigen-specific antibodies. This finding highlights the potential of these CPPs as effective vehicles for delivering antigens [405].

Recently, a study was conducted to characterize two new CPPs derived from the capsid protein (Cap) of the beak and feather disease virus (BFDV), which belongs to the Circoviridae family. The peptides were designated as CPP1 (26 RRYRRRRRYFRKRR 39) and CPP2 (11 RRRRYARPYYRRR 23). The internalization of both peptides was dose- and time dependent, but their absorption efficiency varied depending on the cell type. Their cellular internalization was found to involve multiple pathways, including endocytosis and direct translocation. Both CPPs were able to deliver green fluorescent protein (GFP) and the pc-mCherry, pc-Rep, and pc-Cap plasmids, whose proteins encoded by these plasmids were subsequently expressed in recipient cells successfully. Additionally, CPP1 and CPP2 were able to effectively trigger apoptosis by delivering the apoptin gene, confirming their potential as delivery vehicles [406].

After the identification of the first case of COVID-19 in December 2019, the World Health Organization declared the coronavirus disease of 2019 a pandemic. COVID-19 was caused by a new coronavirus, initially designated as 2019-nCoV and subsequently named severe acute respiratory syndrome coronavirus 2 (SARS-CoV-2). The collected data indicated that we were dealing with a highly infectious virus with a high ability to penetrate cells. Since the virus’s genomic sequence became publicly available, multiple groups have recognized it as a significant source of new CPPs. CPPs could be used to carry pharmacological cargoes or could act as bioactive peptides on their own. In this context, Hemmati et al., using a comprehensive computational approach, identified potential regions of cell penetration in the SARS-CoV-2 proteome. They discovered approximately 310 CPPs, including cationic, amphipathic, and hydrophobic sequences. The critical characteristics of these peptides were verified for their development as drug carriers or biotherapeutic agents, such as antimicrobial and anticancer compounds. The researchers conducted theoretical evaluations of their absorption efficiency, physicochemical properties, solubility, half-life, toxicity, immunogenicity, potential for red blood cell lysis, susceptibility to proteases, inherent and membrane-induced secondary structure, amphipathicity, and lipid-binding potential [407]. In vitro studies are essential to validate the activity of the identified CPPs and accurately assess their antibacterial, antifungal, antiviral, or anticancer properties. Before considering these peptides as potential drug delivery vectors, it is crucial to evaluate their absorption, distribution, metabolism, excretion, and toxicity (ADMET), and initiate studies to determine their stability and effectiveness.

CPPs derived from viral proteins are increasingly being studied to improve the cell permeability of cargo molecules. Among the viral sources of CPPs, Xentry (LCLRPVG) and X-pep (MAARLCCQ) are derived from the X-protein of the hepatitis B virus [408], and VG-21 (VTPHHVLVDEYTGEWVDSQFK) is derived from the vesicular stomatitis virus glycoprotein [409]. These viral CPPs serve as examples of facilitators for the cellular uptake of cargo. Moreover, several viral-derived CPPs have undergone experimental validation, including NLS-A (MTYPRRRFRRRRHRPRS) from porcine circovirus 2 (PCV2) [410], FHV coat-(35-49) (RRRRNRTRRNRRRVR) from the flock house virus (FHV) [100,411], peptides corresponding to the C-terminal domain of Erns from the classical swine fever virus (CSFV) [412], and chimeric Pep1 (KETWWETWWTEWSQPKKKRKV) derived from the simian virus (SV40) [162]. Many of the mechanisms of action for these CPPs have been analyzed using theoretical methods. Additionally, most of these CPPs have been validated for cell permeability using at least one cell line.

### 3.2. CPPs Derived from Animal Venoms and Toxins

Animal venoms and toxins contain pharmacologically active peptide components used by venomous and poisonous organisms for defense and predation. So far, native peptides with cell-penetrating properties have been identified in a limited range of species, including insects (bees and wasps), arachnids (spiders and scorpions), fishes, amphibians, and snakes (elapids and pit vipers). These CPPs exhibit various biological activities, such as toxigenic, cytotoxic, and antimicrobial effects [413]. Numerous studies have explored the potential applications of these peptides in treating pathological processes involving specific interactions with cell membranes or receptor binding. These peptides can translocate through cell membranes and target subcellular compartments, making them suitable for biomedical applications and as potential biopharmaceuticals.

#### 3.2.1. Maurocalcine (MCa)

Maurocalcine (MCa) is the first demonstrated example of an animal toxin peptide with efficient cell-penetration properties. MCa (GDCLPHLKLCKENKDCCSKKCKRRGTNIEKRCR) is a cationic peptide derived from the venom of the Tunisian scorpion *Scorpio maurus palmatus*. In solution, MCa folds to adopt the canonical ICK/knottin motif, in a three-strand arrangement (βββ) constrained by three disulfide bonds. MCa activates the ryanodine receptor (RyR), leading to the rapid release of calcium ions from the sarcoplasmic reticulum in skeletal muscle cells [364]. To achieve this, MCa enters cells, accumulating in both the cytoplasm and nucleus. Its cellular uptake is facilitated by interactions with negatively charged lipids on the cell membrane. MCa has been found to transport various molecules into cells, including the chemotherapy drug doxorubicin [414,415]. To optimize its properties for drug delivery, modified versions of MCa were created, including smaller derivatives without disulfide bonds [416]. Despite these modifications, the version of MCa with native disulfide bonds remained stable in the bloodstream when injected into mice [417].

#### 3.2.2. Imperatoxin A

MCa shares roughly 82% of its sequence with Imperatoxin A (IpTxA, GDCLPHLKRCKADNDCCGKKCKRRGTNAEKRCR), a 3.7 kDa scorpion toxin from *Pandinus imperator venom* [418]. IpTxA activates Ca^2+^-release channels/ryanodine receptors (RyRs) and stands out from typical scorpion toxins and insect defensins by lacking a common consensus motif and deviating from the conventional α/β scaffold. It possesses a cluster of positively charged, basic residues concentrated on one side of the molecule, which may interact with cell membrane phospholipids. In a study focused on understanding how charged amino acid residues in IpTxA affect the gating of RyR1, the authors conducted experiments using synthetic wild-type IpTxA and mutants with alanine substitutions [419]. These mutant toxins were tested on RyR1 incorporated into planar lipid bilayers. Notably, mutants with modified basic amino acids (such as K19A, K20A, K22A, R23A, and R24A) exhibited a significant reduction in substrate production within RyR1. This finding corroborates earlier research suggesting that the essential basic domain of the toxin is responsible for its binding to the channel [419,420]. Furthermore, the domain containing these basic residues, which plays a role in substrate production, shares structural similarities with both MCa and Peptide A [421]. This observation implies a common function of highly concentrated positive charges in influencing the gating of the RyR1 channel. Alternatively, IpTxA could serve as a valuable tool for pinpointing crucial regulatory domains that impact channel gating and for analyzing how skeletal-type RyRs contribute to the intracellular Ca^2+^ waveforms generated by various RyR isoforms upon stimulation [422]. IpTxA has the potential to transport large, membrane-impermeable cargo across the cell’s outer membrane, which has promising implications for innovative drug delivery methods [423].

#### 3.2.3. Melittin

Melittin (GIGAVLKVLTTGLPALISWIKRKRQQ), a cationic CPP, is the principal component of European honeybee (*Apis mellifera*) venom. It adopts an amphipathic α-helix structure characteristic of membrane-interacting and lytic peptides. At low concentrations (lower than 70 μM), melittin inserts into neutral lipid bilayers and phospholipid membranes, while at higher concentrations, it forms transmembrane pores (70 μM). Due to its membrane-disrupting and cytolytic properties, melittin exhibits broad-spectrum anti-infective and anticancer activities [424,425,426,427]. Various melittin derivatives and analogs have been developed to enhance druggability and specific cell membrane penetrability. For example, a chimeric peptide combining melittin with a pro-apoptotic peptide has shown promising results in inducing apoptosis in tumor-associated macrophages, thereby reducing tumor growth [428,429]. Additionally, modified melittin-derived peptides containing Arg and His residues have demonstrated the inhibition of cell proliferation and metastasis, as well as the efficient delivery of siRNAs into the cytoplasm [430]. Truncated versions of melittin have been shown to enhance endosomal escape and transfection efficiency in eukaryotic cells. Moreover, the N-terminal fragment of melittin has been highly effective in facilitating the cellular uptake of nanocrystals and large proteins. These findings suggest the potential of converting cytolytic venom peptides like melittin into safe and effective therapeutics for various biomedical applications.

#### 3.2.4. Anoplin

Anoplin (GLLKRIKTLL) is an amphipathic, α-helical antimicrobial peptide isolated from the venom of the Japanese solitary wasp *Anoplius samariensis*. Anoplin carries a positive charge and interacts directly with negatively charged biological membranes, leading to the formation of an α-helix that disrupts the lipid bilayer. Due to its simple structure and diverse biological and pharmacological functions, numerous research teams have investigated modifications to enhance its effectiveness and availability [431]. For instance, to enhance anoplin’s bactericidal properties by stabilizing its helical structure, Wojciechowska et al. designed and synthesized peptide analogs incorporating hydrocarbon staples. These analogs exhibited improved antimicrobial activity compared to the unmodified peptide. The peptides’ effectiveness varied against Gram-negative and Gram-positive bacteria depending on the staple’s location. The results demonstrated that anoplin’s charge, amphipathicity, and hydrophobic residue positioning influence its ability to disrupt cell walls, affecting its antibacterial activity. Furthermore, the introduced molecular staple increased proteolytic resistance by stabilizing the helical secondary structure [432]. Similarly, Stergiou et al. focused on developing and testing new antibiotic compounds derived from anoplin. By strategically modifying specific amino acids (Leu3 and Arg5 in the interphase, and Thr8 in the polar phase) and attaching fatty acids (substitutions at the N-terminus with octanoic and decanoic acid), the researchers created several anoplin variations. Some of these compounds demonstrated strong activity against both Gram-negative and Gram-positive bacteria, with the most promising compound showing a minimum inhibitory concentration (MIC) value of 0.5 μg/mL [433]. These findings suggest that targeted modifications in the anoplin sequence, along with fatty acid attachments, could yield effective new antimicrobial peptides [434,435,436,437,438].

#### 3.2.5. Mastoparan

Mastoparan (INLKALAALAKKIL) is a cationic peptide extracted from the venom of the wasp *Vespula lewisii*, comprising up to 50% of the venom’s content. Mastoparan and related peptides have served as foundational structures for designing various CPPs, including the mitoparan series and transportans [439,440]. Among these, Transportan (GWTLNSAGYLLGKINLKALAALAKKIL), a 27-residue chimeric peptide combining the N-terminal sequence of the neuropeptide galanin with the 14-residue sequence of mastoparan, has been pivotal in developing a novel series of CPPs known as PepFects and NickFects. Notable examples include PF6 [441], PF14 [442], NF51 [26], and NF55 [443]. A key distinction between traditional CPPs and PepFect or NickFect vectors is that the latter form nanoparticles when loaded with oligonucleotide cargos [444]. Peptides derived from mastoparan exhibit significant potential for various therapeutic applications and promise in biomedical research. Mitoparan (INLKKLAKLAbiKKIL), a mastoparan derivative with structural modifications to reduce cytotoxicity and enhance pharmacodynamic properties, demonstrates greater potency than mastoparan. It functions as both a secretagogue and a cytotoxic agent, efficiently translocating across mammalian cell membranes and targeting mitochondria to trigger apoptosis-mediated cell death [445].

#### 3.2.6. Lycosin-I and Lycosin-II

Lycosin-I (RKGWFKAMKSIAKFIAKEKLKEHL) is a linear cationic peptide derived from the venom of the *Lycosa singorensis* spider. It activates the mitochondrial death pathway, inducing apoptosis in tumor cells, and upregulates p27 to inhibit cell proliferation [446]. When interacting with lipid membranes, it adopts an amphiphilic α-helix structure [447]. Numerous studies have demonstrated lycosin-I’s antibacterial properties and its ability to inhibit tumor growth [435,448]. Notably, lycosin-I induces apoptosis and impedes the migration of prostate cancer cells [449]. Furthermore, it can enter the cytoplasm of tumor cells, initiating signaling pathways that lead to reduced cell proliferation and cell death [446]. However, its potential as a new anticancer medication is limited due to challenges in cell penetration and the effective targeting of solid tumors. To enhance its inhibitory effects on cancer cells, a new peptide variant has been developed. This variant involves replacing lycosin-I’s original lysine with arginine (R-lycosin-I: RGWFRAMRSIARFIARERLREHL), aiming to improve its binding affinity to cell membranes and overall bioavailability. This peptide demonstrated greater anticancer activity and better penetrability against solid tumor cells compared to lycosin-I. Notable distinctions were observed in their physicochemical properties, including secondary structure, hydrodynamic size, and zeta potential [450].

Another peptide similar to lycosin, lycosin-II (VWLSALKFIGKHLAKHQLSKL), displays potent bacteriostatic effects on drug-resistant bacterial strains isolated from hospital patients. Lycosin-II effectively suppressed the growth of all tested bacterial strains, with MIC values ranging from 3.1 to 25 μM, depending on the specific bacteria. Among these strains, *Staphylococcus saprophyticus*, *S. epidermidis*, *Viridans streptococci*, *S. aureus*, and *A. baumannii* showed the highest susceptibility to lycosin-II, while *Klebsiella pneumoniae* and *Streptococcus pyogenes* demonstrated lower sensitivity to the peptide. Notably, significant inhibitory responses were observed only at the highest concentration (50 μM) of lycosin-II. These findings suggest that lycosin-II holds potential as a leading candidate in the development of innovative antibiotics for effectively treating drug-resistant bacterial infections [451].

#### 3.2.7. Pardaxins

Pardaxins (P1 to P5) represent a group of potent ichthyotoxic peptides released into seawater by Moses sole fishes of the genus Pardachirus (*P. marmoratus* and *P. pavoninus*). These peptides share a common structure consisting of a single-chain acidic peptide composed of 33 amino acids, rich in aspartic acid, serine, glycine, and alanine, and devoid of arginine, tyrosine, and tryptophan. Their three-dimensional structure includes an N-terminal hydrophobic α-helix connected to a C-terminal amphiphilic α-helix through a dipeptide (SerPro) [452,453,454]. Pardaxins (GFFALIPKIISSPLFKTLLSAVGSALSSSGGQE) typically insert into biological membranes, creating pores that result in cytolysis [455]. Numerous studies have demonstrated that these effects are achieved through the hydrophobic and pore-forming characteristics of pardaxins, which can cause the lysis of both healthy and tumor cells. For instance, in vitro experiments have shown that pardaxin exhibits antitumor activity against human fibrosarcoma (HT-1080) cells and epithelial carcinoma (HeLa) cells. Importantly, at a concentration of 15 μg/mL, pardaxin did not induce the lysis of human red blood cells. Furthermore, pardaxin dose-dependently inhibited the proliferation of HT1080 cells and induced programmed cell death in HeLa cells [456]. Uen et al. demonstrated that pardaxin can induce the differentiation of leukemic cells into macrophage-like cells with immunostimulatory functions, such as phagocytosis and superoxide anion production. In leukemic THP-1 and U937 cells, pardaxin significantly reduced cell viability and arrested the cell cycle at the G0/G1 phase [457]. These data suggest that pardaxin could be a potential candidate for leukemia treatment, although further studies are required to establish it as a promising therapeutic agent.

Another group demonstrated the anticancer activity of pardaxin in two distinct types of ovarian cancer cells: PA-1 (teratocarcinoma) and SKOV3 (adenocarcinoma). Pardaxin triggers a cytotoxic mechanism by inducing the overproduction of reactive oxygen species (ROS) in mitochondria. This leads to mitochondrial membrane depolarization, resulting in an imbalance in mitochondrial membrane potential and the subsequent activation of pro-caspases 9 and 3. The induction of mitochondria-mediated apoptosis by pardaxin is further supported by the upregulation of t-Bid and Bax. The authors concluded that pardaxin induces excessive mitophagy and mitochondria-mediated apoptosis in human ovarian cancer through the generation of ROS [458].

Additionally, pardaxin has been shown to exhibit anticancer activity both in vitro and in vivo, inhibiting the proliferation and growth of oral squamous cell carcinoma (OSCC). Cell viability assays and colony formation tests on OSCC cell lines (SCC-4) demonstrated that pardaxin reduces cell viability in a dose-dependent manner. Trials for cleaved caspase-3 in SCC-4 cells revealed a significant increase in activated caspase-3 expression after a 24-h treatment with pardaxin. Furthermore, cell cycle analysis indicated that pardaxin treatment led to cell cycle arrest in the G2/M phase, thereby inhibiting cell proliferation in SCC-4 cells. In the 7,12-dimethylbenz[a]anthracene (DMBA)-induced hamster buccal pouch model, pardaxin treatment significantly reduced prostaglandin E2 (PGE2) levels and alleviated carcinogenesis. These results suggest that pardaxin holds potential as a drug for adjuvant chemotherapy in human OSCC and oral cancer [459].

As discussed earlier, CPPs derived from animal venoms have a broad spectrum of applications in biomedicine and biotechnology. These applications encompass diagnostics for detecting diseased cell populations or tissues, as well as therapies that induce selective cell death by delivering drugs or organic compounds that would not typically enter cells. Consequently, discoveries related to venom-derived peptides with cell-penetrating abilities will remain valuable contributions to both basic and applied research in the rational design of CPPs derived from venomous animal components.

## 4. Types of CPP Attachment to Cargo: Covalent and Non-Covalent Strategies with Recent Advances

CPPs are versatile vectors capable of delivering a wide array of therapeutic cargos, including proteins, nucleic acids, and small molecules, across cellular membranes. The method by which CPPs attach to their cargo significantly impacts the efficiency, stability, and functionality of the delivery process. Broadly, CPP–cargo complexes are established through two primary strategies: covalent conjugation and non-covalent complexation. Each approach offers unique benefits and is selected based on the required stability, release mechanism, and nature of the cargo. Below, we explore these strategies in detail, with insights from recent advancements in the field.

### 4.1. Covalent Conjugation: Stability and Targeted Release

Covalent conjugation involves forming stable chemical bonds between the CPP and cargo, ensuring proximity and integrity throughout delivery. This method is frequently used in applications that demand controlled release and stability during systemic circulation. Covalent attachments can be categorized as either non-cleavable or cleavable, each with distinct functional implications.

#### 4.1.1. Non-Cleavable Conjugation

In non-cleavable systems, the bond between CPP and cargo remains intact even after cellular entry, providing a sustained interaction that enhances intracellular retention. This approach is commonly achieved through recombinant DNA technology, where CPPs and cargos—often proteins—are expressed as a fusion protein, or by chemical ligation [460,461]. However, non-cleavable attachment may restrict the activity of the cargo due to the persistent linkage with the CPP, a limitation particularly relevant for bioactive proteins [461].

#### 4.1.2. Cleavable Conjugation

Cleavable conjugates are designed to release the cargo in response to specific intracellular conditions, such as changes in pH or redox potential, which allows targeted action within the cell. Common cleavable bonds include disulfide linkages that are reduced by intracellular glutathione and pH-sensitive linkers that respond to the acidic environments in endosomes and lysosomes [460,461]. For instance, disulfide-linked CPP conjugates allow efficient cargo release within the cytosol, thus preserving cargo bioactivity upon cellular entry [462]. Maleimide linkers, meanwhile, are sensitive to pH changes and can release the cargo selectively in acidic tumor microenvironments, enhancing the specificity of therapeutic action [463].

Recent advancements emphasize the development of bifunctional linkers for CPP–drug conjugates, which have become prominent due to their ability to provide tumor-specific release. These linkers, such as succinyl and acid-sensitive maleimide linkers, facilitate the controlled release of the drug specifically within the tumor, minimizing off-target effects and enhancing therapeutic efficacy. CPP–drug conjugates utilizing bifunctional linkers have shown promise in improving the selectivity and potency of anticancer therapies, establishing a controlled delivery mechanism that responds to environmental stimuli within cancer cells [463].

### 4.2. Non-Covalent Complexation: Flexibility and Adaptability

Non-covalent complexation forms CPP–cargo complexes through weaker interactions, such as electrostatic or hydrophobic forces, without the permanency of a chemical bond. This approach provides flexibility in cargo attachment and can simplify the production process, which is particularly advantageous for sensitive biomolecules like nucleic acids.

#### 4.2.1. Electrostatic Interactions

CPPs with a high content of positively charged residues, such as arginine and lysine, can form electrostatic complexes with negatively charged cargos like DNA or siRNA. This interaction facilitates membrane translocation while preserving the cargo’s structural integrity, making it suitable for nucleic acid delivery [462].

#### 4.2.2. Hydrophobic Interactions and Adaptor Complexes

Hydrophobic cargos benefit from the amphipathic properties of certain CPPs, enabling close contact during delivery through hydrophobic bonding. Additionally, adaptor strategies, such as avidin–biotin coupling, enhance complex stability while allowing cargo release inside the cell. Despite these benefits, non-covalent complexes can be prone to dissociation, particularly within the bloodstream, necessitating careful design to maintain complex integrity during transit to the target site [461].

### 4.3. Innovations in CPP–Drug Conjugation Techniques

Emerging approaches emphasize the use of CPPs conjugated with metal complexes such as ruthenium, which improves both the water solubility and nuclear localization of therapeutic agents. Such CPP–metal conjugates have shown high efficacy in vitro, particularly in cancer cell lines, where they reduce toxicity to non-cancerous cells [463]. Metal–CPP conjugates represent an evolving area of research, offering a unique mechanism for enhancing selectivity and potency in anticancer applications.

**Figure 2:**
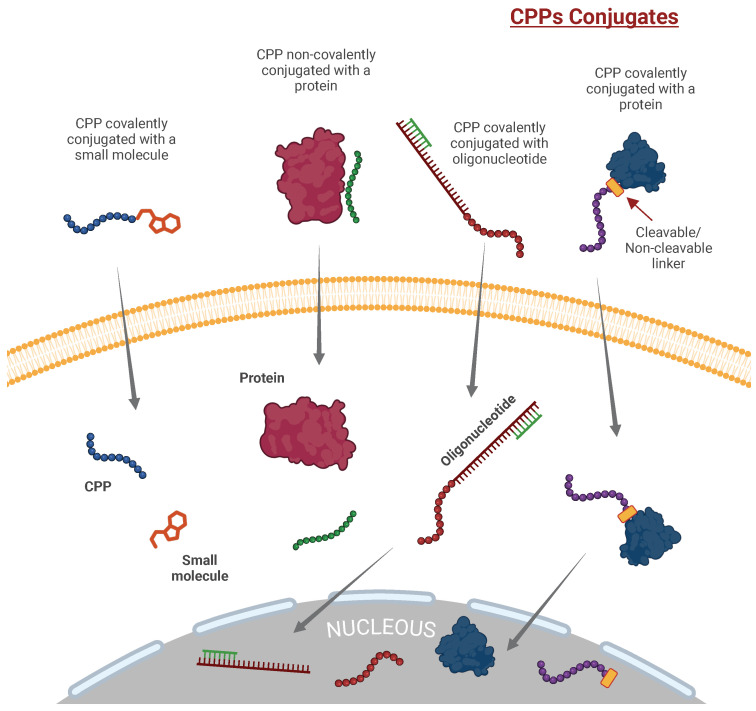
Attachment methods of CPPs to cargo molecules. This figure illustrates two main strategies for CPP–cargo attachment: covalent and non-covalent coupling. Covalent attachment, widely used for intracellular delivery, involves amide, disulfide, or triazole linkages, often with spacers to optimize CPP–cargo distance, and may include fusion proteins for delivering proteins or peptides. Non-covalent attachment leverages electrostatic interactions between positively charged CPPs and anionic cargo or polyanionic carriers. Although less stable in biological environments, non-covalent complexes form easily by simple mixing, as seen with CPP-siRNA interactions. Both approaches offer specific advantages and limitations in terms of stability, specificity, and intracellular delivery efficacy. [Created in BioRender. Moreno-Vargas, L. (2024) BioRender.com/j76w245 | CC-BY 4.0].

Figure 2 illustrates these attachment modalities, contrasting the robust stability of covalent conjugation—exemplified by fusion proteins and chemically ligated compounds—with the adaptable yet transient nature of non-covalent complexes, which are particularly advantageous for delivering sensitive biomolecules, such as nucleic acids.

In summary, advancements in CPP–drug conjugation techniques have expanded the scope for specific and controlled cargo release, tailoring conjugation strategies to meet the environmental conditions unique to tumor cells. These innovations leverage the stability of covalent bonds and the adaptability of non-covalent complexes, positioning CPPs as promising vehicles in targeted drug delivery. Through continued optimization of hybrid approaches and responsive linkers, CPPs hold significant potential for developing safer and more effective therapeutics for a range of diseases, particularly in oncology.

## 5. Cell Translocation Mechanisms of CPPs

While many CPPs share certain properties, particularly their cationic nature, it is widely acknowledged that the uptake mechanisms vary across different CPP families. Most CPPs employ two or more internalization pathways, contingent upon the specific experimental conditions [10]. Numerous studies have proposed that CPPs can enter cells actively through an energy-dependent mechanism or passively via an energy-independent mechanism. One of the primary routes for CPP internalization is endocytosis [464]. In this scenario, CPPs are engulfed by the cell in vesicles or vacuoles pinched off from the plasma membrane, involving distinct steps: interaction with cell surface proteoglycans and/or proteins, followed by endocytic uptake and subsequent endosomal escape [465]. The type of cellular uptake of the CPP depends on several factors, including the physicochemical properties of the peptide, its charge, length, structure, and the applied concentration [466].

Comprehending the influence of various factors is crucial to elucidate the uptake mechanisms of CPPs. In particular, the correlation between the secondary structure of a peptide and its cell penetration capability presents a challenging and intricate aspect. The secondary structure of peptides is profoundly affected by their ambient environment, which plays a pivotal role in determining their efficacy in penetrating cells. This means that peptides can adopt diverse structural forms depending on their location—whether in an aqueous environment, adjacent to or within the cell membrane, or interacting with proteins. Additionally, the importance of a secondary structure in facilitating cellular uptake varies depending on both the type of peptide (cationic, amphipathic, or hydrophobic) and the specific cellular uptake mechanism involved.

For instance, peptides with α-helical and β-strand structures may exhibit heightened sensitivity to mutations that disrupt their three-dimensional conformation. A prominent example of this sensitivity is observed in the amphipathic CPP lactoferrin, which loses both its helical structure and cell penetration efficacy when the disulfide bonds stabilizing its structure are disrupted [467]. Similarly, research on the β-sheet peptide VT5 demonstrated that mutations impairing its β-sheet structure significantly diminished its cellular uptake [468]. Moreover, it is crucial to consider the role of peptide interactions with specific cellular components, such as cell surface receptors, in influencing their ability to penetrate cells. These interactions can play a key role in either facilitating or hindering peptide translocation across cell membranes. Thus, an in-depth analysis of how these interactions affect peptide secondary structure can yield valuable insights. Such insights are essential for advancing our understanding and manipulation of peptide-based cellular penetration in both therapeutic contexts and scientific research.

**Figure 3:**
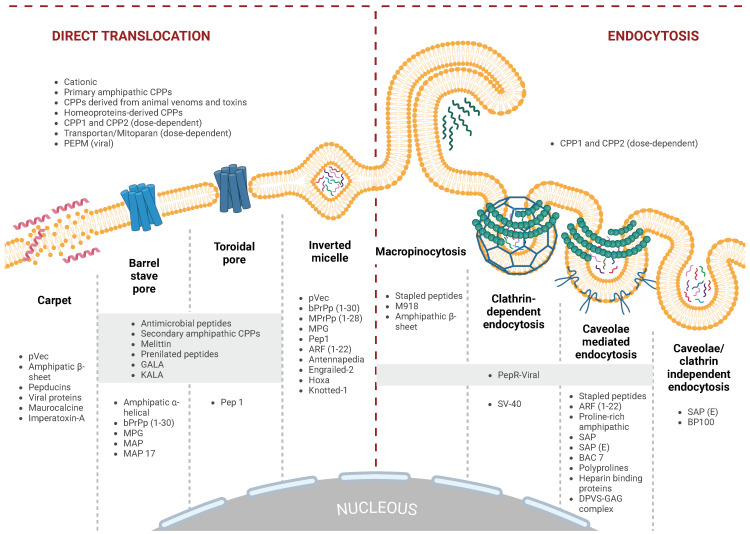
Intracellular entry pathways for CPPs. CPPs utilize two primary mechanisms for cellular entry: energy-dependent endocytosis and energy-independent direct translocation across the lipid bilayer. Endocytosis in this context refers to various internalization processes that can be classified into four major pathways: macropinocytosis, clathrin-dependent endocytosis, caveolae-mediated endocytosis, and clathrin- and caveolae-independent endocytosis. In contrast, direct translocation occurs without energy input and involves mechanisms such as the toroidal or barrel-stave pore formation, inverted micelle, and the carpet model, where peptides disrupt the membrane to facilitate their entry. [Created in BioRender. Moreno-Vargas, L. (2024) BioRender.com/f68s318 | CC-BY 4.0].

The following subsections explore, mechanism by mechanism, the various uptake pathways employed by CPPs. To complement this discussion, Figure 3 provides a schematic representation of these mechanisms, along with a list of known CPPs that use them for membrane penetration.

### 5.1. Direct Translocation

The possibility of CPPs translocating directly through the cell membrane via an energy-independent mechanism, as an alternative to endocytosis, was first suggested when CPP internalization was observed at low temperatures [76]. Direct translocation is considered a single-step process that does not require energy and involves mechanisms such as the formation of inverted micelles, pores, and the ’carpet’ model [469]. Essentially, direct translocation necessitates the interaction of positively charged CPPs with negatively charged components of the cellular membrane, such as the phospholipid bilayer, ultimately facilitating CPP entry. Moreover, direct translocation requires either a permanent or temporary destabilization of the membrane to enable internalization. It is widely acknowledged that direct translocation is more likely to occur at high CPP concentrations (higher than 10 μM) and is most probable for primary amphipathic CPPs, such as transportan analogues and MPG [10,94].

#### 5.1.1. Inverted Micelle Formation

In this type of cellular uptake, the formation of inverted micelles occurs as follows: The first step in the internalization process is the formation of an electrostatic interaction between the peptide and the cell membrane, which affects the supramolecular organization of lipids. This process can lead to changes in membrane curvature. These curvatures or invaginations of the membrane can lead to the formation of inverted micelles that trap the peptide. The hydrophilic environment within the inverted micelle allows for peptide accumulation and is favorable for the transport of hydrophilic compounds conjugated to the peptide. Subsequently, the micelle is destabilized, and the peptide–cargo complex is released into the cytoplasm [10,76,470].

#### 5.1.2. Direct Translocation via Pore Formation

In direct translocation via pore formation, there are two primary models: the ‘barrel-stave’ model and the ‘toroidal’ model. The ‘barrel-stave’ model is characteristic of amphipathic α-helical peptides. These peptides organize into bundles upon interaction with the cellular membrane, forming channels at their centers. The pore is created by the inward-facing hydrophilic surfaces and the interaction between outward-facing hydrophobic residues and the lipid membrane. On the other hand, the ‘toroidal’ model is applicable to peptides that can form α-helices upon interacting with cellular membranes. According to this model, the interaction between the positive side chains of the peptide and the phosphate groups leads to the accumulation of the peptide on the outer leaflet of the membrane. Subsequently, these peptides induce bending of the lipid monolayer towards the interior, creating a hydrophilic gap within the membrane, housing both phospholipid heads and peptides [471,472,473].

#### 5.1.3. Carpet-like Model

In this model, positively charged segments of the peptide align parallel to the membrane surface, binding to the acidic phospholipid headgroups. The peptides self-associate in a ’carpet’-like manner. It is hypothesized that the hydrophobic sites embed into the lipid region of the membrane, while the hydrophilic parts orient towards the hydrophilic region, leading to structural reorganization and internalization of the CPP. Given the necessity of hydrophobic interaction for this model, it appears unlikely to be used for the internalization of strongly cationic peptides. Electrostatic interaction is crucial for the binding between CPP and the membrane. Achieving a high local concentration at the membrane’s surface is also a critical factor for inducing membrane penetration in this model. An alternative model to the ‘carpet’ model is the ‘membrane-thinning’ effect. In this model, a ‘carpet’ formation is initially established, followed by perturbation caused by the interaction between the negatively charged lipids in the outer leaflet of the membrane and the cationic groups of the CPP. This interaction leads to a lateral rearrangement of the negative charges and subsequent thinning of the membrane. The CPPs aggregate on the membrane surface, reducing local surface tension and allowing for CPP intercalation within the membrane. Following peptide internalization, the membrane reseals [10,474,475].

#### 5.1.4. Direct Translocation Mechanisms Used by Arginine-Rich Peptides

Cationic CPPs have been demonstrated to translocate across membranes at low temperatures and in the presence of metabolic or endocytic inhibitors. Experiments using living cells showed that the majority of CPPs are associated with the outer leaflet of the cell membrane. This evidence led to the conclusion that an energy-dependent process is the major route for the internalization of CPPs. Nonetheless, novel studies on living cells show that the uptake of arginine-rich peptides could be a combination of both direct translocation and endocytosis. It has been shown that at low concentrations (below 5 μM), arginine-rich CPPs are mainly endocytosed, whereas rapid cytoplasmic entry occurs at higher concentrations (above 5 μM) [102]. The latter is associated with the accumulation of the peptide at certain membrane areas called nucleation zones [476].

The initial mechanism elucidating the direct penetration of arginine-rich peptides underscores the significance of guanidine groups. It has been demonstrated that oligoarginines can partition into lipid phases from the aqueous phase, especially in the presence of phosphatidylglycerol. The guanidine group in arginine has been shown to form bidentate hydrogen bonds and electrostatic interactions with sulfate, phosphate, and carboxylate moieties—all of which are present on cell surface components. This suggests that the formation of these hydrophobic counterion complexes enhances the accumulation of CPPs on the cell surface and facilitates their internalization. However, during membrane translocation, the peptide backbone must traverse the lipid core. It is hypothesized that hydrophobic interactions between the less hydrophilic peptide backbone and the lipid core are involved in this process [470].

Studies investigating the internalization of polyarginines (R12 peptide) in HeLa cells have revealed a distinct uptake behavior. This behavior is characterized by the formation of structures resembling particles during the interaction and uptake of polyarginines. Both membrane components and R12 contribute to the formation of these “particle-like” structures. The formation and uptake of peptides occur at a low temperature (4 °C) within the first 10–20 min of incubation. The authors propose that these particle-like structures, primarily composed of membrane peptides, lead to membrane inversion and the subsequent absorption of polyarginines. The formation of membrane particles may vary significantly based on the number of arginine residues in the peptide. Specifically, the R12 peptide exhibits a higher affinity for the plasma membrane compared to R8 and R4 peptides, resulting in a more pronounced direct influx [477].

Another mechanism used by arginine-rich peptides to directly traverse cell membranes involves pore formation. Molecular dynamics simulations were conducted to observe the translocation of the cationic peptide TAT, emphasizing the significance of the peptide–phosphate interaction during pore formation. The proposed model suggests that when a specific concentration threshold is met within one membrane leaflet, TAT peptides migrate towards phosphate groups in the opposing leaflet. These peptides collaborate to aid translocation. As the TAT concentration increases, neighboring phospholipids’ phosphate groups are attracted to the peptide due to their opposite charge, ultimately dividing the membrane into regions rich in TAT and phosphate groups. This separation creates uncharged regions, leading to membrane thinning. TAT forms complexes with phospholipids by interacting with negatively charged phosphate groups through arginine and lysine side chains, allowing penetration into the membrane. Concurrently, water molecules infiltrate and solvate the charged groups. Over time, this interaction leads to a transient water pore. TAT then smoothly moves towards the pore walls, transporting attached phospholipids, and successfully crosses the membrane [478].

### 5.2. Endocytosis

It is now widely accepted that CPPs, especially when bound to cargo, are taken up by cells through an energy-dependent process at low concentrations. Endocytosis is an active process, whereby macromolecules are transported into the cell within vesicles or vacuoles pinched off from the plasma membrane. This process involves two distinct steps: endocytic uptake followed by endosomal escape. Endocytosis is a complex process that can be divided into two main types: phagocytosis, which involves the uptake of large particles and occurs in specific cells like macrophages, monocytes, and neutrophils, and pinocytosis, which involves the uptake of fluids and solutes and is a fundamental process in all cells. Pinocytosis includes four distinct mechanisms: macropinocytosis, clathrin-mediated endocytosis (CME), caveolae-mediated endocytosis (CvME), and clathrin- and caveolae-independent endocytosis. Endocytic mechanisms vary based on the cell types and differentiation states, with the choice of pathway influenced by these factors. Additionally, for internalizing carriers like CPPs, their physicochemical properties and surface reactivity are crucial considerations.

#### 5.2.1. Macropinocytosis

Several studies strongly indicate that macropinocytosis is the primary pathway for CPPs to enter cells. Macropinocytosis is a rapid form of endocytosis that does not depend on specific receptors but is dependent on lipid rafts [479]. It occurs when cells are stimulated by growth factors or other signals, leading to membrane ruffling. In this process, actin-driven membrane protrusions extend, resulting in an increased uptake of fluid-phase material [480]. Unlike other endocytic mechanisms where ligand-coated particles are enveloped, macropinocytosis involves the collapse and fusion of these protrusions with the plasma membrane, forming large endocytic vesicles known as macropinosomes [479]. Macropinocytosis is a well-organized process involving significant signaling events that lead to cytoskeleton remodeling. Key regulators of macropinocytosis include kinases (e.g., Src, PI3) and GTPases (e.g., the Rho family, Ras family, and Rab proteins) that drive the formation of actin-driven membrane protrusions [481].

#### 5.2.2. Clathrin-Mediated Endocytosis (CME)

The molecular mechanisms governing clathrin-mediated endocytosis (CME) have been extensively studied, making it the most well-understood type of endocytosis to date. CME is a receptor-dependent process that requires clathrin and dynamin [479]. CME is a fundamental cellular process found in all mammalian cells, playing a crucial role in continuously facilitating the uptake of vital nutrients and intercellular communication during tissue and organ development. In CME, a ligand binds strongly to a specific receptor on the cell surface, initiating the assembly of clathrin proteins into a lattice-like structure on the inner side of the cell membrane. This binding causes the membrane to fold inward, forming a coated pit. Initially, these pits are shallow and progress into dome-like structures that remain connected to the plasma membrane by a funnel-shaped rim. Further invagination leads to the formation of a spherical bud, with the rim transforming into an hourglass-like neck made of membrane. Ultimately, this neck undergoes fission, a critical step facilitated by dynamin, a type of GTPase. After fission, the resulting clathrin-coated vesicles (CCVs) are released and promptly shed their clathrin coats. These uncoated vesicles then travel to early endosomes, which subsequently mature into late endosomes characterized by their low pH environment. The late endosomes transport their cargo to lysosomes, the final destination in this uptake process. CME also regulates the levels of surface signaling receptors and rapidly clears and downregulates activated signaling receptors. Additionally, it plays a significant role in maintaining cell and serum homeostasis by governing the internalization of membrane pumps that control the transport of small molecules and ions across the plasma membrane [482]. CME involves five stages, each intricately coordinated by molecular interactions: (i) the initiation of endocytic events, (ii) cargo loading, (iii) membrane bending, (iv) vesicle scission, and (v) the disassembly of the coat. Each of these stages is highly orchestrated by a series of molecular interactions [470].

#### 5.2.3. Caveolae-Mediated Endocytosis (CvME)

Caveolae are flask-shaped membrane invaginations primarily found on the plasma membrane. These structures owe their shape and structural organization to caveolin proteins, particularly members of the caveolin gene family: caveolin-1 (Cav-1), caveolin-2 (Cav-2), and caveolin-3 (Cav-3). Cav-1 and Cav-2 are widely expressed across various cell types, such as fibroblasts, adipocytes, endothelial cells, and pneumocytes. In contrast, Cav-3 is expressed independently and is primarily confined to skeletal muscle and cardiac myocytes [483]. Among these caveolin proteins, Cav-1 stands out as a key player in shaping caveolae. This small integral membrane protein is characterized by hydrophobic amino acids that insert into the inner leaflet of the membrane bilayer in a hairpin-like fashion. The cytosolic region of Cav-1 serves as a scaffolding domain and is associated with binding to membrane domains rich in cholesterol and sphingolipids. When embedded in the inner leaflet of the plasma membrane, Cav-1 self-associates to create a striated caveolin coat on the surface of these membrane invaginations [484,485]. Notably, Cav-1 exhibits limited mobility at the plasma membrane, contributing to the stabilization of these invaginations’ association with the membrane. This phenomenon delays dynamin-dependent budding and detachment, thereby regulating constitutive endocytosis.

In most cells, caveolae are slowly internalized, a process that takes more than 20 min. The binding of different ligands to caveolin/caveolae, the cross-linking of caveolar components, and the accumulation of receptors within caveolae promote downstream signaling events, ultimately leading to caveolar internalization. Caveolae-mediated endocytosis is highly regulated and, like phagocytosis and macropinocytosis, driven by the cargo molecules themselves [486]. However, the exact molecular mechanisms connecting cargo molecules, caveolae-localized receptors, and the triggered endocytosis process are still not fully understood. Caveolae serve a critical role in delineating regions of the plasma membrane enriched in cholesterol and sphingolipids. These microdomains are instrumental in concentrating a diverse array of signaling molecules and membrane transporters.

Caveolae formation involves proteins other than caveolins. A distinct group of proteins, known as cavins, plays a pivotal role in this process. Unlike caveolins, cavins are peripheral membrane proteins that interact with the molecular components of the caveolar domain facing the cytosol. One key player in this process is PTRF (polymerase I and transcript release factor), also known as cavin-1, which is recruited to the plasma membrane and serves as a coat protein for caveolae. Its binding to the domain containing oligomerized caveolins, cholesterol, and phosphatidylserine stabilizes the membrane curvature, resulting in the characteristic flask-shaped structure of caveolae [484].

#### 5.2.4. Clathrin- and Caveolae-Independent Endocytosis

Lipids and lipid–protein interactions play a crucial role in the functional compartmentalization of the plasma membrane into microdomains or lipid domains. These domains, formed through the interaction of sterols and sphingolipids, give rise to lipid rafts. Lipid rafts diffuse freely over the cell surface; consequently, the division of certain macromolecules into lipid rafts facilitates their internalization through an endocytic pathway independent of both clathrin and caveolae.

Although the mechanisms governing clathrin- and caveolae-independent endocytosis are not fully understood, it is known that this coat-free pathway can be dynamin dependent or -independent. Research has demonstrated that fluid-phase internalization persists even in the presence of a dominant-negative dynamin mutant, which effectively inhibits traditional endocytic mechanisms. This suggests that alternative, dynamin-independent pathways are capable of facilitating the cellular uptake under these conditions [470,479]. The interleukin-2 receptor (IL-2R) is a well-characterized example of a receptor internalized via the dynamin-dependent pathway. Studies have demonstrated that IL-2R subunits localize to lipid raft domains, where dynamin mediates vesicle scission from the plasma membrane. Additionally, actin cytoskeletal elements contribute to the subsequent internalization process, facilitating the receptor’s entry into the cell [479].

Recent evidence suggests that, in the absence of a specific coat protein, lipid accumulation alone can drive membrane deformation, leading to vesicle budding. Clathrin- and caveolae-independent endocytosis may involve not only dynamin but also actin or alternative dynamin-independent mechanisms, such as the ARF-mediated pathway, which has been implicated in SV40 virus entry. Studies on SV40 indicate that this pathway is cholesterol dependent and involves coat-free vesicles characterized by neutral pH and rapid uptake kinetics [487].

As highlighted by Ruseska, flotillin-mediated endocytosis represents another dynamin- and coat-independent pathway as demonstrated by the internalization of GPI-anchored CD59 in HeLa cells. Flotillins 1 and 2 induce membrane invagination in a dose-dependent manner, with phosphorylation by tyrosine kinases likely activating this process. Some evidence also suggests that flotillins may contribute to dynamin-dependent pathways by acting as adaptor proteins for specific cargo [470].

The internalization of CPPs is influenced by their intrinsic physicochemical properties and the characteristics of the membranes they traverse. This internalization process varies across different CPP families, highlighting the diversity of the molecular mechanisms involved. Significant research efforts, including molecular dynamics (MD) simulations, have aimed to clarify the peptide/lipid membrane interactions that promote CPP internalization [62,488,489,490]. However, a comprehensive understanding of the underlying molecular mechanisms, particularly regarding membrane binding and penetration dynamics of CPPs, is yet to be achieved. Take, for instance, the cationic peptide BP100 (KKLFKKILKYL), notable for its strong antimicrobial effects and low hemolytic activity. MD simulations suggest that lysine side chains may play a role in stabilizing the BP100 monomer’s insertion into the membrane through a mechanism known as ‘snorkeling’. Nonetheless, the precise mechanisms by which BP100 and similar antimicrobial peptides disrupt membranes are not fully understood. Experimental studies have demonstrated that BP100 aligns horizontally on the membrane surface at high temperatures in saturated phosphatidylcholine lipids. Near the lipid phase transition temperature, BP100 tends to orient itself vertically, spanning the membrane. This orientation remains stable in thinner bilayers at lower peptide concentrations, while thicker membranes require higher concentrations for similar stability. Intriguingly, even at higher temperatures, BP100 maintains its inserted orientation in the presence of lysolipids [491,492].

Despite these advancements, the complete molecular picture of CPP internalization, including aspects of membrane binding and penetration, continues to be elusive. Integrating a variety of complementary research methodologies is essential for identifying the critical factors that influence CPP cellular entry, thereby facilitating the rational design of efficient molecular transporters.

## 6. Computationally-Aided Design and Prediction of New CPPs

Since the first reports in the literature [2,3], the number of experimentally characterized CPPs has increased rapidly. This growing dataset has allowed researchers to investigate the physicochemical and structural properties that distinguish CPPs from other peptides, with the goal of predicting a peptide’s ability to cross cellular membranes [493]. As a result, not only are these efforts crucial for designing novel CPPs but they have also provided a broader perspective on the common ingredients that enable these peptides to penetrate cellular membranes.

The pioneering attempts to answer these questions came from Hallbrink et al. in 2005 [494] and Hansen et al. in 2008 [495]. Both teams worked with the z-descriptors proposed by Sandberg et al. [496]. These z-descriptors, or z-values, are dimensionless values derived from partial least squares and principal component analysis of physicochemical properties from 87 amino acids. Essentially, z-values capture the key physicochemical differences between amino acids in a reduced-dimensional space. Hallbrink and colleagues examined the average z-values for each peptide in a set of 53 CPPs reported in the literature at the time. By defining specific ranges where these CPPs fell, they reported a prediction success rate of 90% for the CPPs they studied, as well as for 25 non-CPPs. Although this approach might seem simple, they successfully confirmed the cell-penetrating ability of peptides predicted as positive from randomly selected protein segments.

Later, Hansen et al. [495] reported a similar approach using 66 CPPs found in the literature. And shortly after, in 2010, Dobchev et al. [497] introduced the first machine learning predictor for CPPs: an Artificial Neural Network (ANN) trained and tested on a dataset of 52 CPPs and 10 non-CPPs, and validated on a separate set of 25 CPPs and 5 non-CPPs, all with experimental evidence. Despite working with a relatively small CPPs dataset, they achieved an accuracy of 83% in their predictions.

The race that followed in the search for better CPP predictors was vertiginous [493]. A variety of machine learning algorithms have proven efficient in predicting CPPs. In 2011, Sanders et al. developed a predictor based on Support Vector Machines (SVMs) [498], an approach later adopted by Fu et al. [499] and Tang et al. [500,501]. Holton et al. published the CPPpred predictor, using N-to-1 neural networks [502]. The Random Forest (RF) approach was explored by several teams, including Chen et al. [503], Wei et al. with SkipCPP-Pred [504], and Qiang et al. with CPPred-FL [505]. Pandey et al. introduced KELM-CPPred, a predictor based on Extreme Learning Machines [506], while Arif et al. developed TargetCPP using the Gradient Boost Decision Tree (GBDT) algorithm [507]. Liu et al. explored multiview TSK fuzzy systems via HSIC [508], and Rodrigues et al. implemented XGBoost with CSM-Peptides [509]. Most recently, Maroni et al. tested the LightGBM algorithm in their LightCPPgen predictor [510].

Other researchers explored improvements in prediction efficiency by combining different machine learning algorithms. MLCPP, developed by Manavalan et al. [511], employed Random Forests (RFs), Support Vector Machines (SVMs), Extremely Randomized Trees (ERTs), and k-Nearest Neighbors (k-NNs), while MLCPP 2.0 [512] tested a new architecture using SVM, RF, AdaBoost (AB), Light Gradient Boosting (LGB), Gradient Boosting (GB), XGBoost, and ERT. Similarly, CellPPDMod by Kumar et al. [513] utilized RFs, SVMs, Sequential Minimal Optimization (SMO), J48, and Naive Bayes, and Fu et al.’s StackCPPred [514] integrated eXtreme Gradient Boosting (XGBoost), Light Gradient Boosting Machine (LightGBM), KNN, RFs, and SVMs as a meta-classifier.

BChemRF-CPPred, developed by Lima de Oliveira et al., employed an ANN, Gaussian Process Classifier (GPC), and SVM [515], while PreTP-EL by Guo et al. combined SVMs, RFs, and a genetic algorithm [516]. More recent predictors combining ML algorithms are TP-MV and TPpred-ATMV by Yan et al. [517,518], DeepCPPred by Arif et al. [519], and TriplEP-CPP by Serebrennikova et al. [520], among other noteworthy approaches [493].

These earlier predictors differ not only in their choice of machine learning algorithms but also in the input features they utilize. The performance of different algorithms as classifiers depends significantly on the input data. In the case of CPP prediction, machine learning models work by identifying patterns and features specific to CPPs. To achieve accurate predictions, these models must be trained to effectively correlate the selected features with a peptide’s classification as a CPP or a not-CPP.

Initially, the features were based on the z-descriptors proposed by Sandberg et al. [496], which are linear combinations of physicochemical properties, such as molecular weight, side chain van der Waals volume, logP (octanol/water partition coefficient), total accessible molecular surface area, and others (26 features in total). As the field started to advance, Dobchev’s predictor became the first to incorporate features derived from peptide conformations of minimized structures [497]. Building on this, Sanders et al. utilized 61 physicochemical descriptors, including peptide length, charge, weight, and amino acid composition, as well as the percentage of polar and hydrophobic amino acids, secondary structure, steric bulk, and net hydrogen bond donors [498].

CellPPD represented another milestone by integrating dipeptide composition alongside features from the AAindex database [521]. Subsequent predictors greatly expanded the number of features. For example, Chen’s predictor incorporated 400 features, including PseAAC (pseudo-amino acid composition) for the first time [503], while C2Pred also utilized 400 features, focusing on dipeptide frequency to account for sequence information [501]. CPPpred-RF extended this even further to a total of 636 features [522], while CellPPDMod utilized a remarkable 15,537 descriptors [521].

More recent predictors have added even more specialized feature sets. These include composition–transition–distribution (CPPred-FL [505], MLCPP 2.0 [512]), unique motif-based features (KELM-CPPpred [506]), and residue pairwise energy content matrices (StackCPPred [514]). Other predictors used k-mers and top-n-grams (PreTP-EL [516], TP-MV [517], PreTP-Stacks [523]), BLOSUM62 indices (CSM-peptides [509]), and adaptive k-skip-2-g features (SkipCPP-Pred [504]), adding another layer of complexity to the evolving CPP predictors landscape [493].

With the rise in more complex datasets, researchers began exploring deep learning techniques to overcome limitations in feature engineering and capture more intricate patterns [493]. In 2021, Cai et al. introduced ITP-Pred, which utilizes a CNN-BiLSTM model to test this approach [524]. A year later, Zhang et al. developed SiameseCPP, applying natural language processing (NLP) techniques through a Siamese Neural Network combined with fully connected (FCN) layers [525].

Other examples of deep learning-based predictors include PrMFTP by Yan et al. [526], DeepTPpred by Cui et al. [527], AiCPP by Park et al. [528], and PractiCPP by Shi et al. [529]. It is important to note that deep learning approaches do not rely on predefined features; instead, they learn feature representations automatically, and in some cases, they may utilize pretrained models.

These predictors are useful not only for identifying novel CPPs but also for extracting the most relevant features that explain the cell-penetrating ability of these peptides. Many of the characteristics highlighted by the predictors discussed in this section are related to the amino acid composition, charge distribution, and physicochemical properties associated with the hydrophobicity and lipophilicity of the peptides [493]. For example, early predictors revealed physicochemical attributes such as lipophilicity, steric bulk, and polarity [494,495], as well as topographic electronic indices, differences in the charged partial surface area, hydrogen bond donors and acceptors, and the water-octanol partition coefficient [497,498].

Regarding the importance of specific amino acids, Sanders et al. emphasized the role of positively charged residues such as lysine, arginine, and histidine, as well as aromatic amino acids. Similarly, the authors of CellPPD highlighted the significance of residues like Arg and Lys in any position; and specifically Trp, Leu, Ala, and Ile at the N-terminus; and Leu, Ser, and Pro at the C-terminus [521]. Complementing these findings, SkipCPP-Pred identified frequent dipeptides in CPP sequences, including RR, KR, KK, LR, and MM [504]. While traditional machine learning methods rely on predefined features, deep learning models take a different approach. These models typically analyze common patterns in peptides predicted as CPPs, without predefined feature selection. For example, both DeepTPpred [527] and PractiCPP [529] found that amino acids such as Arg, Leu, Trp, and Lys are highly likely to be present in these peptides.

Other physicochemical descriptors following this trend have been uncovered by more recent predictors. For instance, models developed by Chen [503], ITP-Pred [524], CSM-peptides [509], and Multi_CycGT [530] revealed descriptors such as effective partition energy (hydrophobicity), T/T/D charge, conjoint k-spaced triads with n-gaps, cyclohexane-to-water transfer energy, amino acid composition in membrane-spanning regions of proteins, and MlogP (a partition coefficient indicating lipophilicity).

It is worth mentioning that some predictors were designed not only to identify CPPs, but also to assess whether their uptake efficiency is high or low. This is the case for several models, including CellPPD [521], CPPpred-RF [522], MLCPP [511], MLCPP 2.0 [512], and DeepCPPred [519]. These predictors aim to provide not just a binary classification but a deeper insight into how efficiently CPPs penetrate cells, which is crucial for their potential therapeutic applications.

On the other hand, some models were developed with very specific use cases in mind. For example, Wolfe et al. created a predictor tailored to identifying CPPs capable of delivering charge-neutral antisense oligonucleotides, such as phosphorodiamidate morpholino oligonucleotides [531]. Meanwhile, Cao et al. developed Multi_CycGT, a specialized tool designed to predict cyclic CPPs [530]. These focused approaches highlight the growing diversity of CPP prediction tools, each adapted to particular challenges within the field.

Finally, researchers have also attempted to predict CPPs alongside other therapeutic peptides. Diener et al. developed in 2016 a model to predict AMPs and DNA-binding peptides in addition to CPPs [532]. Similarly, Wei et al. introduced the PEPred-Suite, which can predict a variety of peptide types, including antiangiogenic peptides, antibacterial peptides, anticancer peptides, anti-inflammatory peptides, antiviral peptides, CPPs, quorum sensing peptides, and surface-binding peptides [533].

Other valuable predictors have been developed to handle a wide range of therapeutic peptides, including anticoronavirus peptides, antidiabetic peptides, anti-endo-toxin peptides, antifungal peptides, anti-HIV peptides, antihypertensive peptides, anti-MRSA peptides, antiparasitic peptides, antitubercular peptides, blood–brain barrier peptides, biofilm-inhibitory peptides, dipeptidyl peptidase IV peptides, and tumor homing peptides, in addition to those mentioned earlier, including CPPs. Some key models include TP-MV, TPpred-ATMV [518], CSM-Peptides [509], PrMFTP [526], PreTP-Stack [523], and DeepTPpred [527], among others [493].

To conclude this overview of CPP predictors, it is important to acknowledge that the development of machine learning and deep learning-based models has not been without its critiques and areas for improvement. CPPs, after all, are far from a homogeneous group. They exhibit a wide range of chemical features, utilize different cellular mechanisms, and are assessed using diverse experimental methods to measure their uptake efficiency. This complexity often stands in contrast to the predominant approach of training predictors as binary classifiers, either CPP or non-CPP.

One significant area of untapped potential is predicting more nuanced properties, such as different uptake mechanisms or the ability to deliver specific cargos. Although some models have moved beyond simple binary classification, continued development in this direction could greatly enhance our understanding and broader application of CPPs. Furthermore, the lack of unified standards for comparing and evaluating these predictors remains a notable challenge. Establishing a curated, standardized database of CPPs and non-CPPs, together with a consistent set of evaluation tests and metrics, would lay the groundwork for more reliable and comparable models [493].

Despite these challenges, numerous studies in the recent literature demonstrated the successful use of these predictors. For example, CPP predictors have been employed to assess the cell-penetrating potential of the following: antimicrobial peptides isolated from Capsicum annuum [534], the LL-37 human antimicrobial peptide as a therapeutic against SARS-CoV-2 [535], and peptides with the antimicrobial properties found in goat and sheep milk, as well as feta cheese [536].

Additionally, recent work in optimizing and designing novel CPPs demonstrated the potential of these predictors. Some notable examples found in the recent literature include the design of peptides for sustained ocular drug delivery [537], the development of analog peptides derived from Melittin and CXCL14-C17 [538], and the discovery of novel anticancer peptides with cell-penetrating properties targeting lung cancer cells [539].

Ultimately, the remarkable progress made over the past two decades in CPP predictor development highlights the unique and indispensable role these peptides play as therapeutic molecules and drug delivery systems. These advances underscore the versatility of CPPs, and as research continues to push the boundaries of predictive models, CPPs will become increasingly central to next-generation therapeutic strategies.

## 7. Discussion

CPPs share the ability to penetrate membranes, which allows them to be considered members of a single family of therapeutic peptides. However, this family is extremely heterogeneous, with widely diverse physicochemical properties. Based on these properties, they can be categorized into groups such as cationic, amphipathic, hydrophobic, cyclic, or chemically modified peptides. Yet, this variation in physicochemical characteristics is just one aspect of the complexity of this family. In addition to their intrinsic properties, CPPs utilize multiple pathways for cellular uptake, such as macropinocytosis, caveolae/lipid raft-mediated endocytosis, and direct translocation across the cell membrane. And these pathways can also be influenced by external factors, including peptide concentration, cell type, temperature, or pH.

In this rich and intriguing scenario, the role of specific features and interactions that explain and characterize the various uptake mechanisms and their efficiencies remain largely unexplored. For instance, how the conformational landscape of different CPPs, whether in bulk conditions or during membrane translocation—alone or assisted—could offer valuable insights into optimizing and designing new peptides, is still an open area for future research. Additionally, the mechanisms and interactions with membrane proteins that assist some of these peptides in crossing the membrane also require further investigation.

Despite—or perhaps because of—their diversity, CPPs serve as versatile vectors capable of traversing cell membranes while preserving cargo integrity, making them essential and full of potential tools in drug delivery systems. Their specificity, selectivity, and biocompatibility present promising prospects for targeted drug delivery, allowing them to address challenging biological targets with high precision. However, the use of these peptides is not without its drawbacks. There remains significant room for improvement. CPPs face hurdles such as rapid renal clearance and low permeability, which can be mitigated by incorporating non-natural amino acids and associating them with carrier proteins to enhance stability and delivery efficacy. Strategies to improve the pharmacological properties of CPPs—such as increasing permeability, reducing proteolysis, and prolonging half-life—are crucial for enhancing their stability and efficacy in vivo. These approaches aim to develop CPPs with greater resistance to proteolytic degradation and optimized pharmacokinetic profiles, ensuring more effective therapeutic delivery.

The vast majority of CPPs found in the literature are derived from natural sources, often discovered as segments of proteins or from animal venoms and toxins. Since their discovery, the number of natural CPPs has grown rapidly, allowing researchers to explore ways to optimize existing CPPs or even predict novel ones. One might think that the wide variety of penetrating mechanisms complicates the rational development of a universal strategy for optimizing, predicting, or designing CPPs. Yet, despite this complexity, new machine learning and deep learning architectures have been steadily developed and tested over the past couple of decades, each one better than the last. While it may be premature to label these efforts as ‘rational’—as we do with the design and optimization of small molecules—these approaches are proving to be both meritorious and useful tools, as demonstrated by the many recently developed CPP predictors.

Current machine learning models for CPP prediction still face significant challenges, particularly due to their reliance on short peptide sequences and the limited availability of training and validation data. To enhance prediction accuracy and reliability, expanding data collection and refining algorithms are essential steps. Future efforts should prioritize exploring new shallow and deep learning paradigms, while also advancing feature extraction techniques. Some largely unexplored areas in this field include the prediction of specific uptake mechanisms and the identification of specific cargos. Without a doubt, the continued evolution of computational methods, especially deep learning frameworks, will play a crucial role in advancing CPP prediction and design.

In addition to these computational efforts, further experimental advances are needed to better understand membrane traversal mechanisms, loading strategies, and molecular target recognition, all of which are critical for improving drug delivery systems. Future research should also focus on developing novel CPPs that target specific tissues and intracellular organelles, improving therapeutic efficiency while minimizing off-target effects. Linking CPPs with targeting ligands, such as antibodies or small molecules, could further enhance their specificity. Moreover, exploring CPPs with inherent therapeutic effects—such as anticancer, antimicrobial, anti-inflammatory, or immunomodulatory properties—represents an exciting and evolving area of research, highlighting the vast potential of the use of CPPs as precise therapeutic bullets (see Table 1).

### Challenges and Limitations in CPP-Based Therapeutics

Although CPPs hold significant promise as therapeutic delivery vectors, their clinical translation is accompanied by numerous scientific and technical challenges. One of the primary hurdles is achieving selective cellular targeting without off-target effects. Due to their intrinsic ability to permeate diverse cell types, many CPPs lack specificity, which can lead to unintended uptake in non-target cells and, consequently, off-target toxicity. Advances in cell-specific CPPs, such as those developed via phage display, ligand conjugation, or tissue-specific promoters, offer a path toward improved selectivity. However, these strategies add complexity to CPP design and necessitate rigorous optimization and validation to maintain therapeutic efficacy [540,541,542].

Another critical challenge is the susceptibility of CPPs to enzymatic degradation. Given their peptide nature, CPPs are prone to proteolysis in biological fluids, such as blood and gastrointestinal environments, which significantly limits their systemic stability and therapeutic potential. Stabilization techniques, including the incorporation of D-amino acids, peptide cyclization, and N-methylation of amino acids, have been investigated and show promise in enhancing resistance to degradation. However, these modifications may reduce cellular uptake efficiency or alter the bioactivity of CPPs, posing a trade-off between stability and functional performance [541,542].

Efficient transport across physiological barriers, particularly the blood–brain barrier (BBB) and intestinal epithelium, presents another major obstacle. Although CPPs facilitate cellular uptake, their ability to traverse these tightly regulated barriers is often limited. Approaches such as coupling CPPs with receptor-mediated transcytosis or modifying peptides for enhanced barrier permeability are under exploration. However, these modifications may elevate immunogenicity and manufacturing complexity, thus requiring further refinement to ensure both safety and feasibility at scale [541,542].

Endosomal entrapment represents an additional significant challenge in CPP-mediated delivery. Many CPP–cargo conjugates are internalized via endocytosis but become trapped within endosomes, where degradation or recycling processes prevent efficient cytosolic release. Research suggests that specific physicochemical properties of CPPs, such as charge distribution and secondary structure, influence their ability to escape endosomes. Techniques to improve endosomal escape, including the integration of endosomolytic agents or designing CPPs that disrupt endosomal membranes, have shown promise. However, these strategies often increase the risk of cytotoxicity, underscoring the need for careful optimization to balance safety and efficacy [540,541].

Lastly, immunogenicity is a concern for certain CPPs, particularly those derived from viral or highly cationic sequences, which can trigger immune responses that limit clinical utility. Efforts to minimize immunogenicity while preserving therapeutic effectiveness include the design of shorter, less repetitive CPPs or the use of naturally occurring, cell-targeting domains. However, these approaches require a careful balance between reducing immune recognition and retaining therapeutic functionality [540,541].

In conclusion, while CPPs offer a versatile and promising platform for drug delivery, overcoming challenges related to specificity, stability, barrier permeability, endosomal escape, and immunogenicity is crucial for their successful clinical application. Addressing these limitations will require innovative peptide design, advanced delivery strategies, and comprehensive preclinical evaluations to optimize both safety and efficacy, paving the way for CPPs in next-generation therapeutic contexts.

## 8. Conclusions

The remarkable diversity in the sequence array and physicochemical properties of CPPs enables them to exhibit a broad spectrum of structures and functionalities. CPPs can be linear, cyclic, cationic, anionic, hydrophobic, hydrophilic, amphipathic, non-amphipathic, random-coiled, α-helical, or β-sheets. This variety underpins the numerous mechanisms they employ to traverse cell membranes, distinguishing them from most peptides. Highly cationic CPPs, characterized by at least eight positive charges, interact with GAGs and primarily enter cells via endocytic pathways. Upon reaching a specific concentration threshold, they can also translocate directly across the membrane. These CPPs typically do not require a specific 3D structure for cellular uptake. Conversely, secondary amphipathic CPPs must partially adopt a helical structure near the membrane interface, presenting their hydrophobic face to the membrane and their hydrophilic face to the solvent. Although the hydrophilic face can contain a variety of residues (cationic, anionic, and polar), the precise requirements for cell penetration by amphipathic CPPs remain to be fully elucidated. Potential factors include specific amino acid compositions or patterns and the ability to form a helical structure suitable for membrane interaction.

Recently, new hydrophobic CPPs with low net charge and lacking amphipathic arrangement have emerged. These CPPs often contain sequences of hydrophobic amino acids or chemical modifications with hydrophobic chains, such as stapled peptides, prenylated peptides, and pepducins. The origin of a CPP can provide insights into its mechanism of entry. Many cationic CPPs were initially identified in heparin-binding proteins, while numerous amphipathic CPPs were designed or derived from naturally occurring amphipathic peptides, particularly AMPs, which have evolved to interact with diverse microbial membranes. Signal peptides represent a rich source of hydrophobic CPPs due to their innate ability to direct nascent proteins to specific cellular organelles. CPPs must undergo extensive evaluation across a range of assays to determine their toxicity, tissue distribution, cell selectivity, solubility, and plasma stability, among other factors. Mapping how different categories of CPPs perform relative to these parameters is crucial to identifying whether observed issues are inherent to a specific CPP category or can be mitigated through chemical modifications that preserve their uptake capabilities.

Despite their potential, several challenges hinder the clinical translation of CPPs. These include a lack of published human studies, issues with oral bioavailability, tissue and organ-specific toxicity, immunogenicity, and stability in vivo. Current strategies to overcome these challenges involve using fusogenic lipids, the “proton sponge” effect, and membrane-disruptive peptides to enhance endosomal escape. Novel CPPs targeting specific tissues and intracellular organelles have been developed to improve therapeutic efficiency and reduce off-target effects. Furthermore, linking CPPs with targeting ligands such as antibodies, folic acid, transferrin, and RGD peptides enhances their specificity. Techniques like the ATTEMPTS strategy protect CPPs from enzymatic degradation and increase their target specificity. Despite these advancements, no CPP or CPP/cargo complex has yet received FDA approval, primarily due to their non-specificity, instability, and suboptimal pharmacokinetics. Ongoing research efforts to address existing limitations, combined with the robust preclinical efficacy demonstrated by CPPs, indicate that CPP-based therapeutics may enter pharmaceutical markets in the medium term. PEP-010, the first therapeutic peptide derived from PEP-Therapy’s CP&IP technology, exemplifies this potential through its selective inhibition of pathological intracellular interactions, exhibiting significant antitumor efficacy and a favorable safety profile in multiple preclinical models conducted by the Curie Institute [543].

CPPs hold significant promise for the diagnosis and treatment of various diseases, including cancer, inflammation, central nervous system disorders, and viral infections. Continued research and refinement in understanding their mechanisms of cellular entry and optimizing their pharmacological properties will be pivotal in harnessing the full potential of CPPs in clinical applications. In conclusion, the extensive structural diversity and versatile nature of CPPs provide a robust foundation for advancing therapeutic delivery strategies. The future of CPPs in clinical applications looks promising, potentially heralding a new era in targeted therapeutic delivery systems.

## Figures and Tables

**Table 1:** CPP-based therapeutics in clinical trials.

CPP	Cargo	Compound	Application	Status	ClinicalTrial.gov ID
Cationic CPPs					
HIV-1 Tat-protein-derived Tat peptide	δPKC inhibitor	KAI-9803	Acute myocardial infarction	Phase II completed 2004	NCT00093197
HIV-1 Tat-protein-derived Tat peptide	MAGE-A3/HPV-16		Head and neck carcinoma	Phase I completed 2005	NCT00257738
HIV-1 Tat-protein-derived Tat peptide	ϵ-PKC inhibitor		Pain: postherpetic neuralgia, spinal cord injury, postoperative	Phase II completed 2010	NCT01106716
HIV-1 Tat-protein-derived Tat peptide	δPKC inhibitor	KAI-9803	Heart attack	Phase II completed 2011	NCT00785954
HIV-1 Tat-protein-derived Tat peptide	ϵ-PKC inhibitor		Pain: postherpetic neuralgia, spinal cord injury, postoperative	Phase II completed 2011	NCT01135108
HIV-1 Tat-protein-derived Tat peptide	Dextrogyre peptide		Intraocular inflammation and pain	Phase I completed 2012	NCT01570205
HIV-1 Tat-protein-derived Tat peptide	Botulinum toxin A		Cervical dystonia	Phase III completed 2012	NCT01753310
HIV-1 Tat-protein-derived Tat peptide	ϵ-PKC inhibitor		Pain: postherpetic neuralgia, spinal cord injury, postoperative	Phase II completed 2013	NCT01015235
HIV-1 Tat-protein-derived Tat peptide	D-JNKI-1 gel		Hearing loss, idiopathic sudden sensorineural	Phase III completed 2015	NCT02561091
HIV-1 Tat-protein-derived Tat peptide	D-JNKI-1 gel		Hearing loss, idiopathic sudden sensorineural	Phase III completed 2016	NCT02809118
HIV-1 Tat-protein-derived Tat peptide	JNK-1	XG-102	Intraocular inflammation and pain	Phase III completed 2016	NCT02235272
HIV-1 Tat-protein-derived Tat peptide	PSD-95 protein inhibitor		Ischemic stroke	Phase III completed 2016	NCT02930018
HIV-1 Tat-protein-derived Tat peptide	Botulinum toxin A		Cervical dystonia	Phase II completed 2016	NCT02706795
HIV-1 Tat-protein-derived Tat peptide	Dextrogyre peptide	XG-102	Postoperative ocular inflammation	Phase III completed 2017	NCT02508337
HIV-1 Tat-protein-derived Tat peptide	JNK-1	AM-111	Acute inner ear hearing loss	Phase III completed 2017	NCT02561091
ATX-101			Advanced dedifferentiated liposarcoma and leiomyosarcoma	Phase II completed 2023	NCT05116683
ATX-101	Carboplatin		Fallopian Tube and Primary Peritoneal Cancer	Phase I/II terminated 2024	NCT04814875
(R-X-R)_4_, X = 6-Aminohexanoic acid	PMO targeted to human c-Myc	AVI-5126	Obstruction of vein graft after cardiovascular bypass surgery	Phase II completed 2009	NCT00451256
TransMTS	Botulinumtoxin A		Cervical dystonia	Phase III completed 2022	NCT03608397
MTS	Botulinumtoxin A		Lateral canthal lines	Phase III completed 2016	NCT02580370
MTS	Botulinumtoxin A		Primary Axillary Hyperhidrosis	Phase II completed 2016	NCT02565732
AVB-620	Tetramethylindo(di)-carbocyanines (Cy5 and Cy7)		Breast cancer	Phase I completed 2015	NCT02391194
AVB-620	Tetramethylindo(di)-carbocyanines (Cy5 and Cy7)		Breast cancer	Phase II completed 2021	NCT03113825
Z12	BI754091	BI754091/ATP128/VSV-GP128	Stage IV Colorectal Cancer	Phase I active, not recruiting, 2024	NCT04046445
R7	Cyclosporine A	PsorBan	Psoriasis	Phase II terminated 2003	Not Applicable
PEP-010	Paclitaxel		Metastatic solid tumor cancer	Phase I recruiting (2024)	NCT04733027
Amphipatic CPPs					
p28	p28 Non-HDM2-mediated peptide inhibitor of p53	p28	Solid tumors	Phase I completed 2014	NCT00914914
p28	p28	p28	Central Nervous System Tumors	Phase I completed 2017	NCT01975116
p28	Valganciclovir (VGCV)	RZ-001	Glioblastoma	Phase I/II recruiting	NCT06102525
p28	SRF388	Atezolizumab/Bevazizumab		Phase II active	NCT05359861
PTD4	AZX100 (a synthetic 24-amino acid peptide analog of heat shock protein 20 (Hsp20))		Excision of Keloid Scars	Phase II completed 2012	NCT00825916
Hydrophobic CPPs: Stapled Peptides					
Sulanemadlin (ALRN-6924)	Cytarabine		Acute Myeloid Leukemia or Advanced Myelodysplastic Syndrome	Phase I completed 2019	NCT02909972
Sulanemadlin (ALRN-6924)	Palbociclib		Solid Tumor/Lymphoma/Peripheral T-Cell Lymphoma	Phase I/Phase II completed 2020	NCT02264613
Sulanemadlin (ALRN-6924)	Topotecan		Small cell lung cancer	Phase I active (Study completion (Actual) 2022)	NCT04022876
Sulanemadlin (ALRN-6924)	Cytarabine		Solid tumor, brain tumor, leukemia or pediatric lymphoma	Phase I active (Study completion (Actual) 2023)	NCT03654716
Sulanemadlin (ALRN-6924)	Doxorubicin/Cyclophosphamide/Docetaxel		TP53-Mutant Breast Cancer	Phase I active (Study completion (Actual) 2023)	NCT05622058
Sulanemadlin (ALRN-6924)	Palbociclib		Advanced, Metastatic, or Unresectable Solid Tumors	Phase I active (Study completion (Estimated) 2025)	NCT02264613
Pepducins					
Pepducin PZ-128 (P1pal-7)			Multiple Coronary Artery Disease Risk Factors	Phase I completed 2016	NCT01806077
Pepducin PZ-128 (P1pal-7)			Coronary artery disease	Phase II completed 2021	NCT02561000
Cyclic CPPs					
^177^Lu-DOTA^0^-Tyr^3^-octreotate	Lutetium Lu 177		Neuroendocrine Tumors	Not Applicable completed 2019	NCT02125474
^177^Lu-DOTA^0^-Tyr^3^-octreotate	Lutetium Lu 177		Neuroendocrine Carcinoma	Phase II active 2023	NCT02236910
BT1718			Solid tumors	Phase I/Phase II completed 2024	NCT03486730

## Abbreviations

The following abbreviations are used in this manuscript:

3D	Three-Dimensional
aCPP	Amphipathic Cell-Penetrating Peptide
ADME	Absorption, Distribution, Metabolism and Excretion
AML	Acute Myeloid Leukemia
AMP	Antimicrobial Peptide
ANN	Artificial Neural Network
Bac	Bactenecin
BCT	Bicycle Toxin Conjugate
BFDV	Beak and Feather Disease Virus
BH3	Bcl-2 Homology 3
BIM	Bcl-2-Interacting Mediator
BIP	Bax-Inhibiting Peptide
BMP	Bis(Monooleoylglycero) Phophate
BPrPp	Bovine Prion Protein
cCPP	Cationic Cell-Penetrating Peptide
cyCPP	Cyclic Cell-Penetrating Peptide
Cav	Caveolin
CAV	Chicken Anemia Virus
CAPH	Cationic Amphiphilic Polyproline Helix
CCV	Clathrin-Coated Vesicle
CD	Circular Dichroism
CME	Clathrin-Mediated Endocytosis
CPP	Cell-Penetrating Peptide
CPP5	Cell-Penetrating Pentapeptide
CSFV	Classical Swine Fever Virus
CTGF	Connective Tissue Grouwth Factor
CTL	Cytotoxic T Lymphocyte
CvME	Caveolae-Mediated Endocytosis
DENV	Dengue Virus
DMBA	7,12-dimethylbenz[a]anthracene
DPVs	Diatos Peptide Vectors
ELP	Elastin-Like Polypeptide
EN1	Neural-specific transcription factor Engrailed 1
ERT	Extremely Randomized Tree
FGF	Fibroblast Growth Factor
FHV	Flock House Virus
GAG	Glycosaminoglycan
GBDT	Gradient Boost Decision Tree
GEP	Gastroenteropancreatic
GFP	Green Fluorescent Protein
GPCR	G Protein Coupled Receptor
hCPP	Hydrophobic Cell-Penetrating Peptide
HSPG	Heparan Sulfate Proteoglycan
HS	Heparan Sulfate
HTX	Hepatocyte transplantation
iPep	Interfering Peptide
IL-2R	Interleukin-2 Receptor
IpTxA	Imperatoxin
k-NN	k-Nearest Neighbor
LGB	Light Gradient Boosting
LightGBM	Light Gradient Boosting Machine
MAP	Model Amphipathic Peptide
MCa	Maurocalcine
MD	Minimal Domain
MDR	Multidrug Resistance
MIC	Minimum Inhibitory Concentration
MPG	N-methyl DNA Glycosylase
MPrPp	Mouse Prion Protein
MT1-MMP	Membrane Type 1 Matrix Metalloproteinase
MTS	Membrane Translocation Sequence
NET	Neuroendocrine Tumor
NLS	Nuclear Localization Sequence
ODN	Oligodeoxynucleotide
OSCC	Oral Squamous Cell Carcinoma
paCPP	Primary Amphipathic Cell-Penetrating Peptide
pHACS	Hemagglutinin Cleavage Site Peptides
PAR	Protease Activated Receptor
PCNA	Proliferating Cell Nuclear Antigen
PCV2	Porcine Circovirus 2
PDX	Patient-Derived Xenograft
PI	Phophatidylinositol
PL	Polylysines
PNA	Peptide Nucleic Acid
PP2A	Protein Phosphatase 2A
PPII	Poly-L-Prolyne Type II Helix
PRRT	Peptide Receptor Radionuclide Therapy
PTD	Protein Transduction Domain
PTRF	Polymerase I and Transcript Release Factor
pVEC	VE-cadherin-derived Cell-Penetrating Peptide
RCM	Ring-Closing Metathesis
RF	Random Forest
ROS	Reactive Oxygen Species
saCPP	Secondary Amphipatic Cell-Penetrating Peptide
SAHB	Stabilized α-Helix of BCL-2 domains
SAP	Sweet Arrow Peptide
SAR	Structure–Activity Relationship
SCLC	Small Cell Lung Cancer
SMO	Sequential Minimal Optimization
SSTR	Somatostatin Receptor
SSTR2	Type 2 Somatostatin Receptor
SVM	Support Vector Machine
TNBC	Triple-Negative Breast Cancer
